# Stability of Metal–Organic Framework-Supported Amines under Exposure to Ozone Generated from Air

**DOI:** 10.1021/acs.iecr.6c01324

**Published:** 2026-07-09

**Authors:** Mario Zorrilla-Valtierra, Botagoz Kuspangaliyeva, Yuhe Cao, Jonas Baltrusaitis, Ryan P. Lively, Krista S. Walton

**Affiliations:** † School of Chemical & Biomolecular Engineering, 6798 Georgia Institute of Technology, 311 Ferst Drive, Atlanta, Georgia 30332, United States; ‡ Department of Chemical and Biomolecular Engineering, 1687 Lehigh University, 111 Research Dr., Bethlehem, Pennsylvania 18015, United States; § Department of Chemical and Biomolecular Engineering, North Carolina State University, 911 Partners Way, Raleigh, North Carolina 27695, United States; ∥ Department of Civil, Environmental and Construction Engineering, University of Texas at El Paso, 500 W University Ave, El Paso, Texas 79968, United States

## Abstract

Amine compounds supported on porous materials such as metal–organic frameworks (MOFs) have shown promising performance for direct air capture (DAC) due to their enhanced affinity for CO_2_. Although features such as adsorption capacity and selectivity are paramount in these composites, their long-term stability has a major impact on the operating cost of DAC systems. In this work, changes in carbon capture performance, crystallinity, porosity and chemical environment of the constituting atoms of MOF-amine composites are explored after exposure to ozone and NOx impurities generated from corona discharge applied to air. From the obtained results, the stabilities of Mg_2_(dobpdc) (dobpdc^4–^ = 4,4′-dioxidobiphenyl-3,3′-dicarboxylate) grafted with ethylenediamine (en), *N*-methylethylenediamine (men), and *N*,*N*-dimethylethylenediamine (dmen), as well as MIL-101­(Cr) MOF impregnated with polyethylenimine (PEI), are compared. A negative effect in the overall CO_2_ adsorption capacity is observed for all MOF composites after exposure, as well as a decrease in the adsorption step pressure of CO_2_ for Mg_2_(dobpdc) amine-grafted composites, as shown via dynamic gravimetric adsorption experiments. Spectroscopic analyses indicate that oxidation of amine groups through the formation of nitro functional groups occurs as well as a decrease in the electron-donation interaction between the supported amines and the metal nodes of the MOFs.

## Graphical abstract

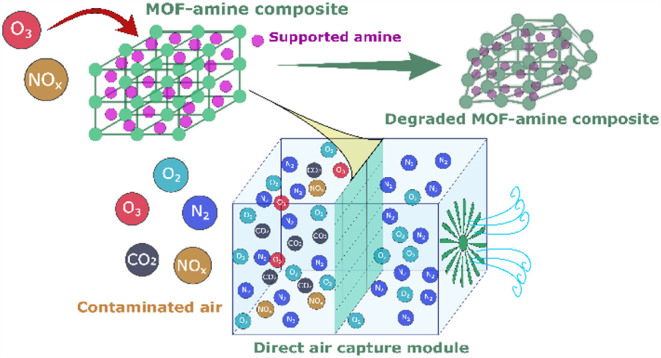

## Introduction

Metal–organic frameworks (MOFs) are compelling for carbon capture applications due to their high porosity and tailorable properties^1^ that enable them to interact with CO_2_ through different adsorption mechanisms.^2^ While CO_2_ capture capacity for DAC can be maximized through techniques such as postsynthetic modification^3^ with organic amines,^4,5^ the successful deployment of DAC processes is highly dependent on operational costs, where the degradation of the sorbent has a significant influence.^6–8^ Indeed, DAC sorbents are expected to be exposed to species that may oxidize amines either during the CO_2_ loading process^9^ or during thermal regeneration.^10^ Additionally, SO_2_ ^11^ and NO_2_ ^12^ can reduce CO_2_ adsorption capacity by either inhibiting or oxidizing supported amines or by promoting the collapse of the support structure.

It is therefore imperative to study the interaction between DAC sorbents and contaminants found in air. Ground-level ozone (O_3_) is a highly oxidative species, which has been found to degrade rubber^13^ and vulcanized isoprene rubber^14^ even when present at ultradilute concentrations in air. However, studies in the literature focused on its effect over supported amine sorbents are almost nonexistent,^15^ and those focused on the effects on MOFs are scarce.^16,17^

In this work, ethylenediamine (en), *N*-methylethylenediamine (men), and *N*,*N*-dimethylethylenediamine (dmen) supported on Mg_2_(dobpdc) and MIL-101­(Cr) supported polyethylenimine (PEI) are exposed to air containing ozone generated from a commercial corona discharge generator. Applying corona discharge to air also generates nitrogen oxide impurities.^18,19^ The effects of ozone and NOx impurities were analyzed through sets of dilute and high-concentration exposures simulating long-term degradation and extreme conditions.

The amines and supports were selected based on literature studies finding Mg_2_(dobpdc)-mmen^20–23^ and MIL-101­(Cr)-PEI^24–26^ promising for DAC applications. However, the chosen *N*,*N*-dimethylethylenediamine isomer has primary and tertiary amines instead of two secondary amines as mmen. The rest of the chosen alkylamines have either a primary and secondary amine (men) or two primary amines (en) with similar alkyl chain lengths. This experimental design allows us to compare the effects of O_3_ and NOx on different terminal amines while keeping the metal-bound amine unchanged, while PEI supported on MIL-101­(Cr) is useful to highlight the effect of ozone and NOx impurities on supported polyamines. Structural degradation, amine modification, and changes in the CO_2_ adsorption performance are analyzed through a combination of spectroscopic and adsorption analyses. Effects of having different amine moieties and adsorption mechanisms on sorbent properties after exposure are discussed.

## Experimental Procedures

### Material Synthesis

#### Chemicals and Gases

All chemicals were used as received. Chromium nitrate nonahydrate (Cr­(NO_3_)_3_·9H_2_O, 99%), magnesium nitrate hexahydrate (Mg­(NO_3_)_2_·6H_2_O; 99%), terephthalic acid (BDC: C_8_H_6_O_4_ (98%), glacial acetic acid (ACS grade, 99.7% purity), and branched polyethylenimine Mw: 800 (PEI) were acquired from Millipore Sigma; 4,4′-Dihydroxy-[1,1′-biphenyl]-3,3′-dicarboxylic acid (H_4_dobpdc, 97% purity) from Ambeed, Inc.; ethylenediamine (en: C_2_H_8_N_2_, 99%) from Thermo Fisher Scientific; and *N*,*N*′-dimethylethylenediamine (dmen: C_4_H_12_N_2_, 98%) as well as *N*-methylethylenediamine (men: C_3_H_12_N_2_; ≥97%) from TCL America. Ultrazero-grade air and ultrahigh-purity nitrogen were purchased from Airgas.

MIL-101­(Cr) was synthesized using a scaled-up version from Darunte et al.^24^ 2 g (∼5 mmol) of Cr­(NO_3_)_3_·9H_2_O and 0.88 g (∼5 mmol) of BDC were mixed while sonicating in 25 mL of deionized water and 3.8 mL of 36% (v/v) aqueous acetic acid and heated to 200 °C for 12 h, followed by cooling to room temperature at 1 °C/min. 2–3 mg of MIL-101­(Cr) were added as seed crystals. The product was centrifuged and washed with water and a 50 mM aqueous solution of NH_4_OH as a lower toxicity alternative to NH_4_F^27,28^ (see Supporting Information Figures S1–S2). Afterward, MIL-101­(Cr) was activated at 150 °C for 12 h under vacuum and kept in a desiccator.

Mg_2_(dobpdc) was synthesized using a scaled-down version from Siegelman et al.^29^ 2.022 g of Mg­(NO_3_)_2_·6H_2_O (7.88 mmol) were added to a pressure vessel together with a filtered solution of 1.25 g (4.56 mmol) of 4,4′-Dihydroxy-[1,1′-biphenyl]-3,3′-dicarboxylic acid (H_4_dobpdc) in 50 mL of 55:45 (v/v) methanol:*N*,*N*-dimethylformamide (DMF) homogenized through sonication. The vessel was sealed and heated to 120 °C in an oil bath for 20 h while stirring. Mg_2_(dobpdc) solids were vacuum filtered and soaked three times in 200 mL of DMF, followed by solvent exchange in triplicate using 200 mL of methanol at 60 °C.

Mg_2_(dobpdc) was activated at 180 °C for 16 h under vacuum.^30^ The vacuum oven was backfilled with N_2_, and Mg_2_(dobpdc) was sealed and kept inside a vacuum desiccator until used. Proof of the conservation of its stability can be found in Supporting Information (Figure S3).

#### Diamine-Appended Mg_2_(dobpdc)

Ethylenediamine (en), *N*-methylethylenediamine (men), and *N*,*N*-dimethylethylenediamine (dmen) were appended to Mg_2_(dobpdc) following a scaled-up version from Parker et al.,^31^ which was modified by activating the MOF at 180 °C before grafting the amines. To illustrate, 30 mL of 20% diamine solution in toluene (v/v) was added together with 180 mg of activated Mg_2_(dobpdc) to a round-bottom flask and stirred at room temperature overnight. Solids were vacuum filtered, washed with toluene over the filter, activated at 120 °C for 12 h under vacuum, and kept in a vacuum desiccator.

#### Polyethylenimine-Impregnated MIL-101­(Cr)

Branched polyethylenimine (PEI) Mw: 800 was impregnated into MIL-101­(Cr) following procedures from literature.^32,33^ MIL-101­(Cr) was sonicated in methanol for 30 min. In parallel, a methanol solution with the stoichiometric amount of PEI to achieve 1 mmol/g loading was stirred for 30 min. Subsequently, both dispersions were mixed and stirred at room temperature overnight. MIL-101­(Cr)-PEI was isolated through rotary evaporation at 40 °C, activated under vacuum at 120 °C for 3 h, and kept in a vacuum desiccator.

### Ozone Exposure Experiments

Amine-loaded MOFs were activated at 110 °C for 3 h; Mg_2_(dobpdc) and MIL-101­(Cr) were activated at 180 and 150 °C, respectively, for at least 12 h before exposure to O_3_ and NOx generated using an MP-8000 generator from A2Z Ozone Inc. In each experiment, 80 mg of sample were loaded onto a 20 mm fritted glass tube from Chemglass, which was attached to the exposure setup shown in Figure 1. Preliminary trials can be found in Supporting Information (Figures S4–S5).

**Figure 1:**
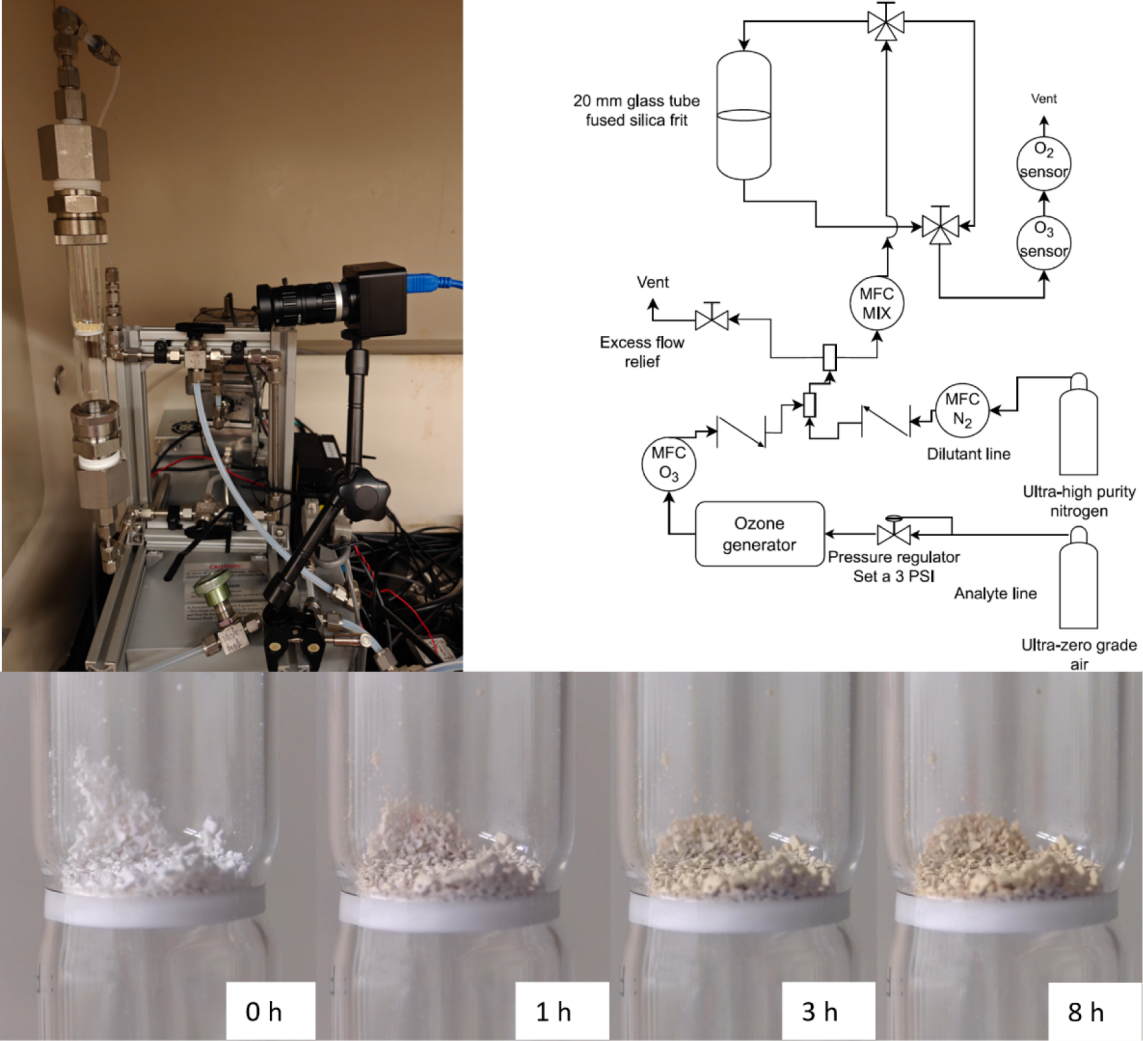
Photograph of ozone exposure unit (top left) and process flow diagram (top right). Change in color of Mg_2_(dobpdc)-dmen during exposure to oxygen-deficient air containing 50 ppm of O_3_ (bottom). Similar color changes are observed for all diamine-appended samples at this condition.

In the first set of experiments, fresh samples were exposed to 50 mL/min of a stream of oxygen-deficient air containing 50 ppm of ozone and NOx impurities in 2% oxygen as measured on OX-B431 and O2-A2 electrochemical sensors from Alphasense Ltd. The samples were exposed for 8 h to simulate a time-weighted average (TWA) of ∼57 ppb-year O_3_, which is below the limit established by the Environmental Protection Agency (EPA) of 70 ppb^34^ in the United States as well as below the limit set by the European Union of 60 ppb.^35^

As stated previously, applying corona discharge to air promotes the generation of nitrogen oxides.^18,36^ Nevertheless, it is expected for the effects of ozone to outweigh those of nitrogen oxides over the amine-supported sorbents based on reported reaction rates between ozone and amines^37^ and those of nitrogen oxides and amines.^38^

The IOX-B431 sensor is also sensitive to NO_2_; however, the main expected byproduct is N_2_O. Thus, we surmise that NO_2_ will not have a significant effect on the sensor’s reading.

For the second set of experiments, sorbents were exposed for 8 h to 50 mL/min of air carrying a higher concentration of ozone by operating the generator at full capacity. The concentration of this second experiment was too high for the sensor to measure, but it is expected to be higher than 1000 ppm, as per A2Z Ozone Inc. specifications. Using air, the ozone generator can produce 6 mg/L of O_3_, which corresponds to 2500–3000 ppm of O_3_.

After exposure, sorbents were reactivated in a vacuum oven at the previously stated temperatures and times, backfilled with N_2_, sealed, and stored in a vacuum desiccator.

### Characterization

#### X-ray Photoelectron Spectroscopy (XPS)

As synthesized and exposed samples were analyzed by XPS. Sorbents were kept under vacuum for at least 6 h after activation, loaded onto a holder with recesses, and compacted as much as possible to achieve a uniform surface for analysis (Figure S6), which allows for minimizing charging effects.^39^ XPS scans were collected using a Thermo K-Alpha spectrometer with a monochromated aluminum Kα source (λ = 8.3386 Å). A spot size of 400 μm was used for all analyses, and the flood gun was used to neutralize charging effects. Survey scans were collected at a pass energy of 200 eV and step size of 1 eV, and core high-resolution scans were collected at a pass energy of 20 eV and step size of 0.1 eV. High-resolution XPS scans were collected at binding energy (BE) ranges for C 1s, N 1s, O 1s, and either Cr 2p or Mg 1s. Dwell time for both survey and core scans was set at 0.05 s. Scofield relative sensitivity factors (RSF)^40^ were used in quantification together with the instrument-measured transmission function and effective attenuation length correction (EAL).^41^ CasaXPS v2.3.6rev1.0Q was employed for all data processing tasks; collected XPS core spectra were referenced by setting the main peak of the C 1s spectra for all samples at a BE of 285.0 eV to account for charging effects.^42^ The inelastically scattered background was subtracted using Shirley’s background.^43^

#### Attenuated Total Reflectance-Fourier Transform Infrared (ATR-FTIR) Spectroscopy

ATR-FTIR spectra of each sample were collected as fresh, after exposure to ozone before reactivation, and after the thermal regeneration after exposure. Spectra were collected using a Nicolet iS50 Spectrometer (ThermoFisher) equipped with an iS50 ATR module. Each analysis consisted of 64 scans with a resolution of 0.4 cm^–1^. Thermal regeneration was performed at 110 °C for 3 h for amine-supported samples, 180 °C for at least 12 h for bare Mg_2_(dobpdc), and 150 °C for at least 12 h for MIL-101­(Cr).

#### Gas Adsorption Isotherms

N_2_ adsorption isotherms were collected at 77 K using a Micromeritics Tristar II Plus surface analyzer using relative pressure dosing, a P_0_ tube to analyze atmospheric pressure, and an equilibration interval of 10 s. Amine-loaded samples were activated for 3 h at 110 °C, and MOF samples were activated at 150 °C overnight. The BET surface area of all samples was calculated using BETSI.^44^ Pore volumes are reported at P/P_0_ = 0.797.

CO_2_ adsorption isotherms were obtained at 25 °C using a Micromeritics 3Flex high-performance gas analyzer through absolute pressure dosing and an equilibration interval of 5 s.

#### Dynamic Adsorption CO_2_ Gravimetric Measurements

Dynamic adsorption experiments were performed at 25 °C using a TA Instruments Thermo Gravimetric Analyzer Q500 (TGA) with the furnace connected to a purge flow of N_2_ and an analysis gas flow of 400 ppm of CO_2_ in N_2_. Approximately, 10 mg of diamine-appended samples were exposed to a flow of 90 mL/min 400 ppm of CO_2_ balanced with N_2_ for 6 h subsequently to being activated at 110 °C under a flow of 90 mL/min UHP N_2_ for 3 h.

#### Powder X-ray Diffraction (PXRD)

Diffraction patterns were collected using a benchtop Rigaku Miniflex 600 Powder X-ray Diffractometer (PXRD) equipped with a Cu Kα radiation source set at 40 kV (λ = 1.542 Å) and a D/tex Ultra detector with a step size of 0.01° and analysis speed of 10°/min. Zero background sample holders were used for all the analyses.

#### CHN Elemental Analysis

Flask combustion CHN elemental analyses were performed before and after exposure after thermal regeneration in duplicate by Atlantic Microlabs Inc.

#### Thermogravimetric Analysis

Thermogravimetric analysis (TGA) was performed before and after exposure to a high concentration of ozone using a TA Instruments TGA 550 and heating from 30 to 600 °C at a rate of 10 °C/min.

## Results and Discussion

### Color Changes during Exposure

At the start of the ozone exposure experiment, the flow exiting the generator was diverted through the bypass to ensure that the concentration would reach 50 ppm. Then, the flow was directed through the exposure cell by switching both the inlet and outlet three-way valves. A camera was adapted to the setup to analyze visual changes in the samples during exposure, as shown in Figure 1. Subtle yellow to brown coloration of diamine-appended Mg_2_(dobpdc) samples became more evident at longer exposure times (Figure 1). Mg_2_(dobpdc) also presented a different color after exposure (Figure S7), while no changes were observed in MIL-101­(Cr) or MIL-101­(Cr)-PEI (Figures S8–S10). Color changes were more evident at shorter times for diamine-appended samples exposed to high ozone concentrations (Figures S11–S13) and for Mg_2_(dobpdc) (Figure S14) compared to the lower concentration exposures (Figure S8). No notable change in color was observed for MIL-101­(Cr)-PEI or for MIL-101­(Cr) after exposure to either diluted ozone or highly concentrated ozone (Figures S15–S20).

### Changes in Porosity of MOF Supports and Amine Composites

BET surface areas (Figure S21) were calculated from the N_2_ isotherms (Figures S22–S26) at 77 K using BETSI.^44^ The pore volume of each sample was obtained from the adsorption isotherm at a relative pressure of P/P_0_ = 0.797. Changes in the porosity of the samples before and after exposure to 50 ppm are summarized in Table 1.

**Table 1:** Cryogenic N_2_ Physisorption (77 K) Porosity Measurements of MOF and MOF-Supported Amines after Synthesis and after Exposure to Oxygen-Deficient Air Containing 50 ppm of Ozone for 8 h

	Property	MIL-101(Cr)	MIL-101(Cr)-PEI	Mg_2_(dobpdc)	Mg_2_(dobpdc)-en	Mg_2_(dobpdc)-men	Mg_2_(dobpdc)-dmen
% change after exposure to 50 ppm of O_3_	BET SA (m^2^/g)	–6.9	+5.6	–12.5	–8.5	–33.2	–51.5
	Vp (cm^3^/g)	–4.3	+5.9	–9.5	–10.0	–32.6	–51.1

Several factors can lead to changes in sorbent porosity. For instance, partial collapse of the structure or pore clogging due to trapped molecules can lead to such loss, which can be assessed by analyzing the diffraction pattern of the materials.^45,46^ As shown in Table 1, the surface area and pore volume of MIL-101­(Cr) and Mg_2_(dobpdc) are only marginally affected after exposure to 50 ppm of ozone.

The change in surface area and pore volume of Mg_2_(dobpdc)-men is more significant compared to that of Mg_2_(dobpdc)-men, and Mg_2_(dobpdc)-dmen porosity is the most affected of the three. This result suggests a different interaction between O_3_ and the grafted amine that could be dependent on the type of terminal amine facing the pore cavity. For instance, the reaction between O_3_ and either primary or secondary amines occurs through hydrogen abstraction in the liquid phase, generating OH radicals and nitro compounds; however, the reaction with tertiary amines has low yields of OH radicals, and it usually produces amine oxides in solution or amine radicals that degrade to secondary amines.^37^

According to the mechanisms of interaction previously mentioned, hydrogen abstraction, generation of water, and production of nitro groups should be more favorable than interaction with tertiary amines, but changes in porosity of Mg_2_(dobpdc)-en suggest that having available primary amines is better for keeping the structural stability compared to having no terminal primary amines as Mg_2_(dobpdc)-dmen. This effect could be related to the findings in the calculations ofJamdade Jamdade et al.,^15^ where the energy barrier for the reaction between secondary amines in *N*,*N*′-dimethylethylenediamine and O_3_ is higher when the number of molecules present in the MOF pore increases.

### Changes in CO_2_ Capture Performance

The exposure of Mg_2_(dobpdc)-men and Mg_2_(dobpdc)-dmen to 50 ppm of O_3_ resulted in a negative shift in the adsorption step pressure^29^ of CO_2_ on their volumetric adsorption isotherms (Figure 2). This shift is potentially due to the presence of electron density-pulling moieties that can weaken the bond between the metal-bound amine and Mg, a point which is later addressed in this manuscript through XPS and ATR-FTIR.

**Figure 2:**
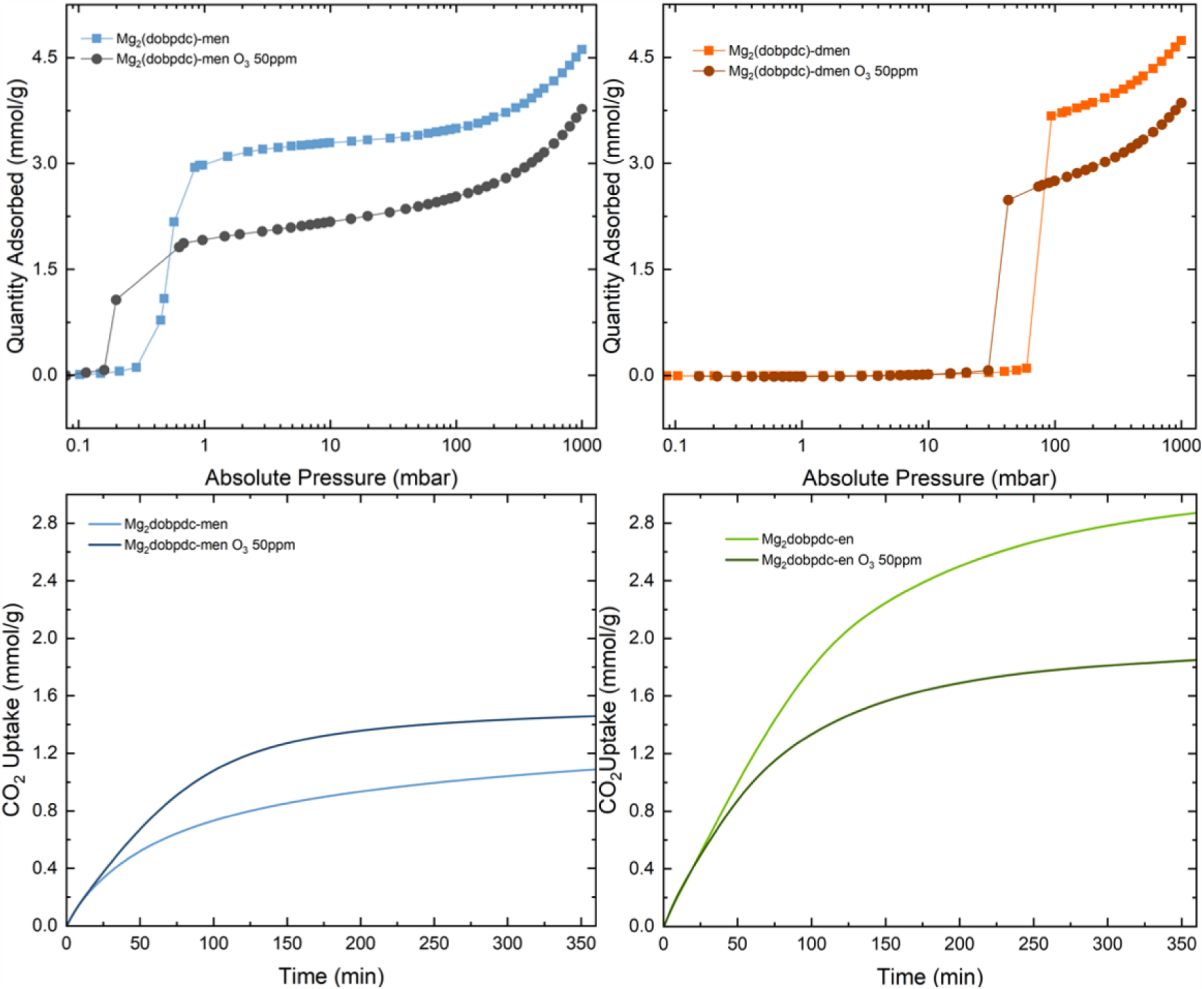
CO_2_ isotherms with logarithmic pressure scale of Mg_2_(dobpdc)-men (top left) and Mg_2_(dobpdc)-dmen (top right) before and after exposure to 50 ppm of ozone. Dynamic adsorption gravimetric measurements with 400 ppm of CO_2_ in N_2_ for Mg_2_(dobpdc)-men (bottom left) and Mg_2_(dobpdc)-en (bottom right) confirming changes in CO_2_ adsorption capacity. Darker colored lines represent samples exposed to 50 ppm of O_3_.

The rest of the samples exhibit a decrease in adsorption capacity within the full range of pressure analyzed (Figures S27–S31). This is expected for samples relying on primary amines for adsorption of CO_2_ due to inhibition of amines through the generation of moieties that are not able to act as Lewis bases and enhance the affinity of the MOFs for CO_2_.^47,48^

To verify the changes observed in the isotherms, Mg_2_(dobpdc)-en and Mg_2_(dobpdc)-men samples were analyzed before and after exposure to 50 ppm of O_3_ through gravimetric dynamic adsorption measurements while flowing 400 ppm of CO_2_ balanced with N_2_ (Figure 2 bottom).

Similar to the results in equilibrium adsorption measurements, the adsorption capacity of Mg_2_(dobpdc)-men is found to be higher compared to that of the fresh sample after exposure to 50 ppm of O_3_. Changes in the chemical composition of the samples are further investigated through XPS and FTIR spectroscopy.

### Effect of Ozone Exposure on Sorbent Structure

PXRD patterns were analyzed for any changes that could indicate structure collapse (Figure 3). The diffraction patterns of Mg_2_(dobpdc)-en and Mg_2_(dobpdc)-men after exposure represent a more crystalline structure compared to that of the fresh samples, which is attributed to the loss of amorphous material. This is also observed for the parent MOF, where the reflections shift to lower diffraction angles and become sharper in the exposed sample’s diffraction pattern. No peak broadening or intensity loss was observed for either MIL-101­(Cr) or MIL-101­(Cr)-PEI (Figure S32).

**Figure 3:**
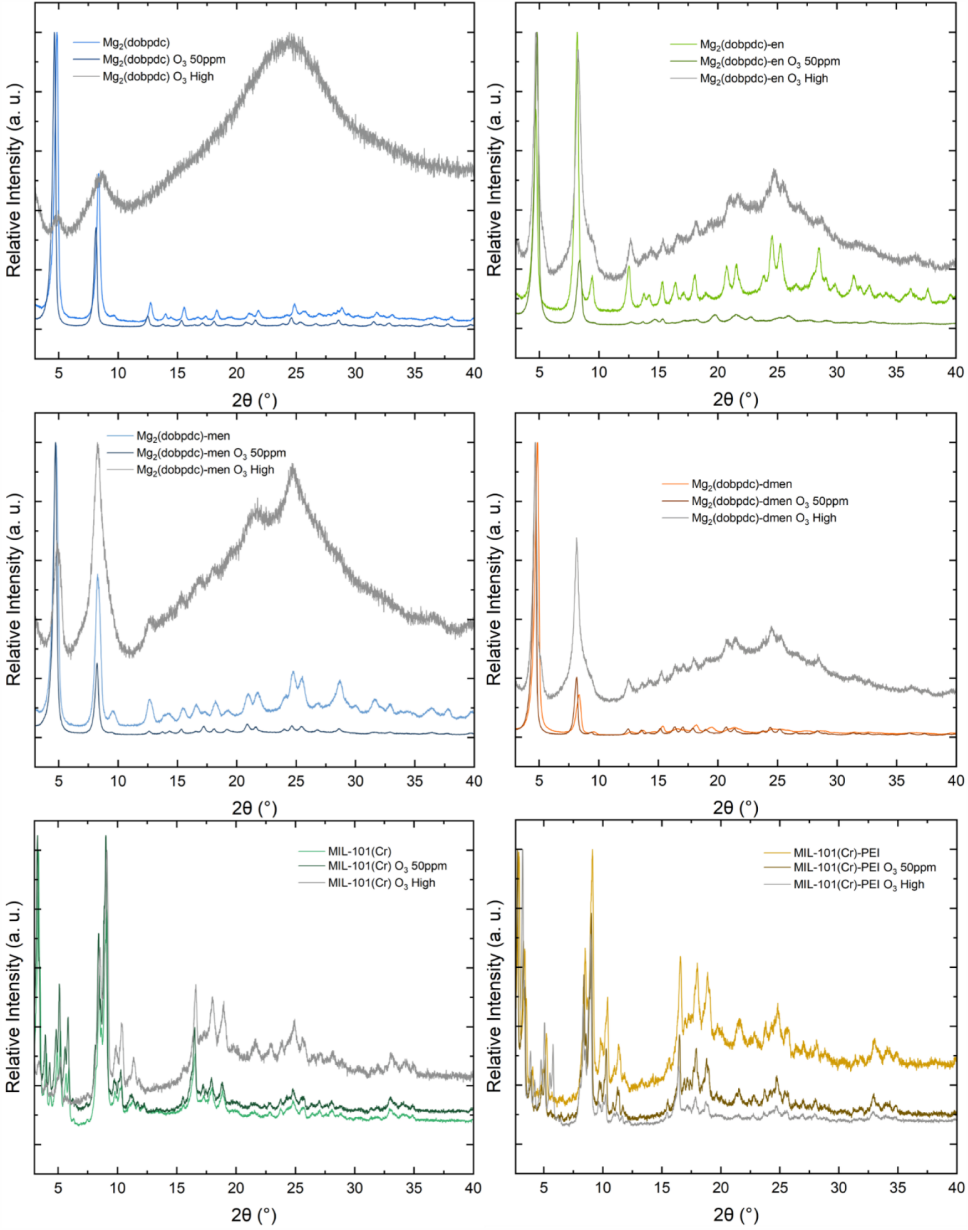
Powder diffraction patterns of bare and amine-decorated Mg_2_(dobpdc) and MIL-101­(Cr) samples before exposure (bright colored lines), after exposure to oxygen-deficient air containing 50 ppm of O_3_ (dark colored lines), and after exposure to air containing high concentrations of O_3_ and nitrogen oxide byproducts (gray lines).

Since no significant changes are observed in the diffraction pattern of diamine-appended exposed samples, it can be inferred that changes in porosity for Mg_2_(dobpdc)-men and Mg_2_(dobpdc)-dmen exposed to dilute ozone are either not due to a significant collapse of the structure or the change in the structure cannot be observed at this extent of degradation.

On the other hand, PXRD analyses of Mg_2_(dobpdc) exposed to high concentrations of O_3_ showed loss of crystallinity, as the reflections of the exposed samples are broader than those of the fresh samples (Figure 3, gray lines). Mg_2_(dobpdc)-men suffers a more significant degradation compared to Mg_2_(dobpdc)-en and Mg_2_(dobpdc)-dmen. Moreover, higher relative intensity of the reflection is found at 4.7°, and sharper reflections are observed for Mg_2_(dobpdc)-dmen compared to Mg_2_(dobpdc)-en and Mg_2_(dobpdc)-men. The most notable change in this reflection is observed for Mg_2_(dobpdc)-men, and the rest of the pattern pertaining to the exposed sample above 10° resembles that of the exposed Mg_2_(dobpdc) MOF with some broad reflections still visible. Based on these observations, the most structurally stable compound would be Mg_2_(dobpdc)-dmen followed by Mg_2_(dobpdc)-en, Mg_2_(dobpdc)-men, and Mg_2_(dobpdc) would be the least stable.

Reflections with lower relative intensities are observed when comparing exposed and fresh MIL-101­(Cr) diffraction patterns. This may indicate that ozone and NOx may promote the rupture of some of the coordination bonds with BDC ligands, which should be a significantly slower process compared to the degradation of Mg_2_(dobpdc). MIL-101­(Cr)-PEI exposed to high concentrations of O_3_ exhibits sharper reflections with higher intensity at low diffraction angles. This suggests either a loss of amorphous material from the PEI-impregnated sample or an increase in free space within the pores that allows the X-rays to penetrate to the MOF support structure of the composite material.

### Spectroscopic Evaluation of Exposed Composites

ATR-FTIR spectra were used to assess the formation of new functional groups and better understand the cause for the change in CO_2_ adsorption performance. Sorbents were analyzed as synthesized and after exposure to ozone before and after thermal reactivation. Spectra were corrected to reduce scattering and any other artifacts using standard normal variate (SNV) correction^49^ (Figures S33–S37). The most evident changes for diamine-appended samples occur in the region 1310–1350 cm^–1^ in the form of a sharp absorbance band for Mg_2_(dobpdc)-en; a broader band is observed for Mg_2_(dobpdc)-men, and no significant changes are observed for Mg_2_(dobpdc)-dmen (Figure S34). The bands observed with 1310–1350 cm^–1^ are related to the N–O symmetric stretch, while those found at 1480–1530 cm^–1^ are representative of N–O asymmetric stretch.^50^

As synthesized and exposed sorbents were analyzed using XPS spectroscopy. After impregnation with PEI, a negative shift in binding energy of ∼1 eV was observed in Cr 2p (Figure S38), while a similar shift of ∼0.4 eV was observed in Mg 1s after diamine grafting. Negative shifts suggest an increase in electron density around the metal nuclei,^51^ which is attributed to the interaction of primary amines present in PEI and diamines with Cr and Mg atoms in MIL-101­(Cr) and Mg_2_(dobpdc), respectively. Negative shifts are also observable in O 1s core spectra (Figure S39). The presence of residual solvent is observed in N 1s scans of MIL-101­(Cr) and Mg_2_(dobpdc) fresh samples (Figure S40), albeit the signal-to-noise ratio is quite low, indicating only a negligible amount.

A model of two peaks is proposed for fitting N 1s core spectra of fresh samples, which accounts for terminal amines facing the pores as C–NR_2_ (R = C or H) as well as metal-bound amine as N—C=O, following results presented in the literature^52^ (Figure 4). After exposure to dilute ozone, the presence of an intense peak at 407 eV in the N 1s core spectra of Mg_2_(dobpdc)-dmen is attributed to the formation of nitrates (NO_3_). These groups would form at the metal-bound amine and pull electron density from it, thus reducing the step pressure for CO_2_ adsorption as observed in Figure 2. An additional peak in N 1s spectra of exposed Mg_2_(dobpdc)-dmen identified as R–NO is found at higher binding energy than C–NR_2_ and N—C=O, which is attributed to partially oxidized metal-bound amine groups since it is centered at lower binding energy than that of NO_2_ of 405.7 eV. Subtle changes are also observed in MIL-101­(Cr)-PEI after exposure to 50 ppm of O_3_ as a small peak forming at 405.7 eV, which is attributed to NO_2_.

**Figure 4:**
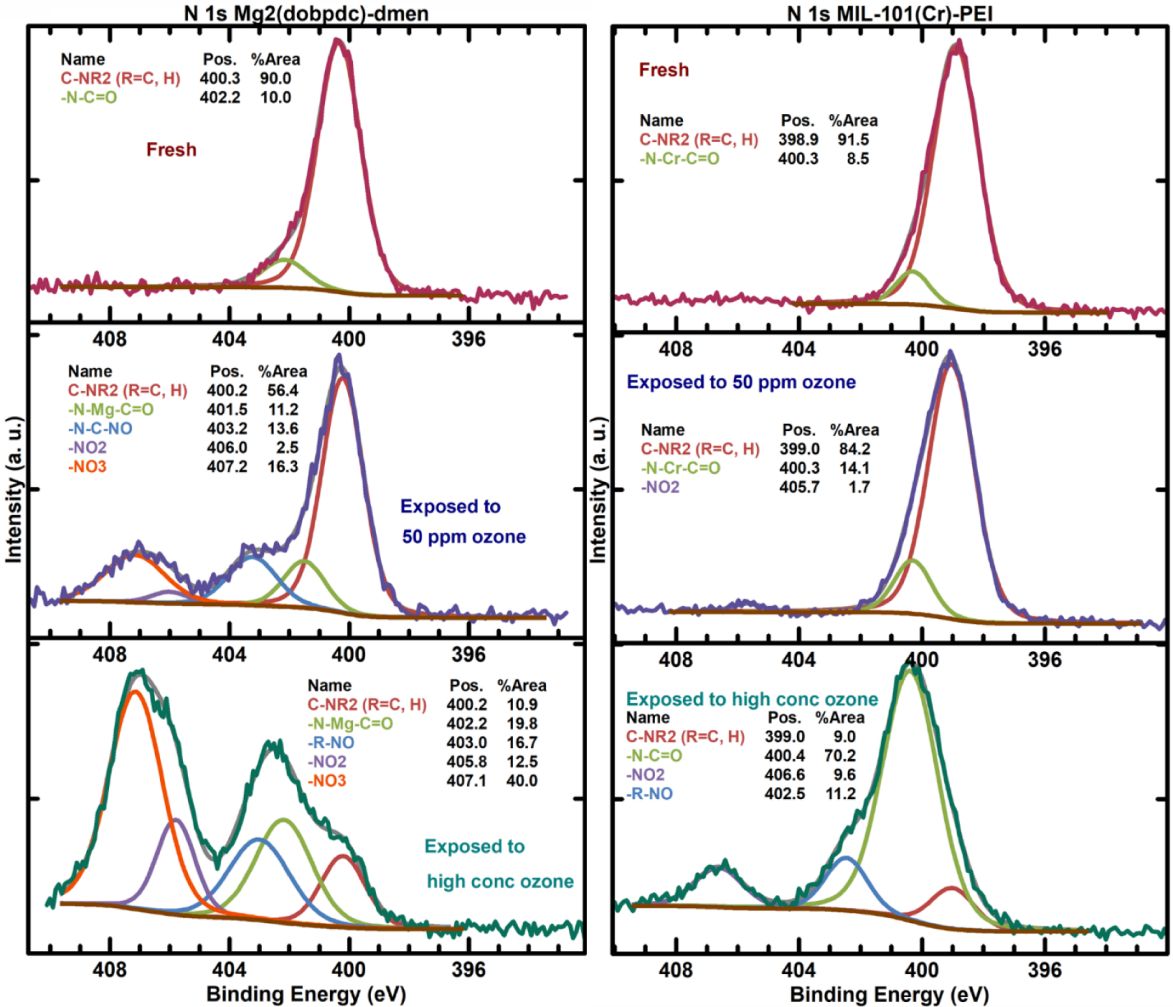
N 1s scans of diamine-appended Mg_2_(dobpdc)-dmen (left) and MIL-101­(Cr)-PEI (right) after exposure to high concentrations of ozone. Exposure to 50 ppm ozone shows a more significant effect for Mg_2_(dobpdc)-dmen than for MIL-101­(Cr)-PEI. XPS Cr 2p scans of MIL-101­(Cr)-PEI exposed to 50 ppm ozone (bottom left) and to highly concentrated ozone (bottom right).

Changes in XPS spectra were not observed for the other diamine-appended samples nor in that of the MOF samples (Figures S41 and S42). On the other hand, abundant nitrogen species were found in Mg_2_(dobpdc) diamine-appended MOFs exposed to high concentrations of ozone. The proposed peak model applied to Mg_2_(dobpdc)-dmen in Figure 4 is used to identify NO_3_ at about 407.1 eV, which is comparable to nitrate at 407.3 eV in MgNO_3_ from the literature,^53^ and NO_2_ at 405.8 eV, while the peak identified as C-NR_2_ attributed to amines is found at 400.3–400.4 eV. The peak centered at 403 eV is proposed to represent amine oxides and potential nitrosoalkanes^37^ as products of amine degradation. A summary of these assignments and references can be found in Table S3.

These nitrogen oxide species were also found in Mg_2_(dobpdc)-en and Mg_2_(dobpdc)-men exposed to high concentrations of ozone (Figure S43). The difference between the proportion of the C–NR_2_ to NO_2_ and NO_3_ products between the exposed Mg_2_(dobpdc)-en and Mg_2_(dobpdc) grafted with men and dmen is attributed to the crowding effect previously mentioned in the CO_2_ adsorption section, where the spontaneity of the reaction between O_3_ and amines seems to be affected by the number of amines available to react with it. Nitrogen oxides were also found in Mg_2_(dobpdc) after exposure to high concentrations of ozone, which suggests the adsorption of NOx impurities and subsequent reaction with ozone (Figure S44). Additionally, an increase in the percentage of C–O and C–N is observed (Figure S45), which suggests an interaction between the H_4_(dobpdc) ligand of Mg_2_(dobpdc), ozone, and NOx impurities, to which the change in color of the sample during exposure is attributed.

In MOF-amine composite samples, a positive shift in binding energy was found in Cr 2p and Mg 1s spectra when comparing fresh samples and those exposed to high concentrations of O_3_ (Figure S46). This shift is attributed to a reduction in the electron donation from primary amines to metal sites in MIL-101­(Cr) and Mg_2_(dobpdc) due to heavy oxidation, as found in ATR-FTIR (Figure S33).

The peak centered at 406.7 eV in MIL-101­(Cr)-PEI exposed to a high concentration of O_3_ is attributed to NO_2_ species. The difference in position is attributed to abundant electron density-pulling moieties such as C=O, which can be observed in the C 1s spectra for exposed MIL-101­(Cr)-PEI (Figure S47).

### Elemental Analysis

The elemental composition of samples exposed to high concentrations of ozone (Table S2) suggests that supported amine samples degrade by reacting with both O_3_ and NOx due to an atomic change ratio of ΔO/ΔN > 2, or an increase in more than 2 atoms of oxygen per atom of nitrogen. Particularly, Mg_2_(dobpdc)-en experiences a ΔO/ΔN higher than 4, which suggests a more favorable interaction between terminal primary amines and O_3_, and a less favorable interaction with NOx. As for MIL-101­(Cr) and Mg_2_(dobpdc), ΔO/ΔN ∼ 2 suggests that adsorption of NOx dominates, and further oxidation of adsorbed species like NO or N_2_O when interacting with O_3_ may occur. These results are supported by CHN elemental analysis (Figure S48), where a substantial increase in N percentage is observed for Mg_2_(dobpdc), while there is a slight increase in exposed amine composites. A loss of carbon wt % is also observed that either suggests degradation and loss of organic compounds or a substantial increase in wt % of oxygen in the samples, which is visible in elemental analysis from XPS scans in Tables S1 and S2. In the case of the MOFs, this result would translate into ligand degradation and loss during thermal regeneration, which would explain the collapse of Mg_2_(dobpdc) as seen from the changes in its diffraction pattern.

Based on the results presented in this work as well as kinetic studies performed by Lim et al.,^37^ MOF-amine composites that rely on cooperative adsorption are more prone to degradation due to interaction with O_3_ and NOx, while these species are expected to barely reach the MOF structure when a polyamine is supported in it (Figure 5). Results from exposing diamine-appended Mg_2_(dobpdc) to 50 ppm of ozone show that terminal primary amines can prevent changes in porosity or reduce the impact of degradation on the structure compared to secondary and tertiary amines. Moreover, Mg_2_(dobpdc)-en does not present products like nitrates that can suggest interaction with the metal-bound amine after exposure to 50 ppm of ozone. Thus, it is more likely for O_3_ and NOx to interact close to metal sites when terminal moieties are not able to interact with these contaminants.

**Figure 5:**
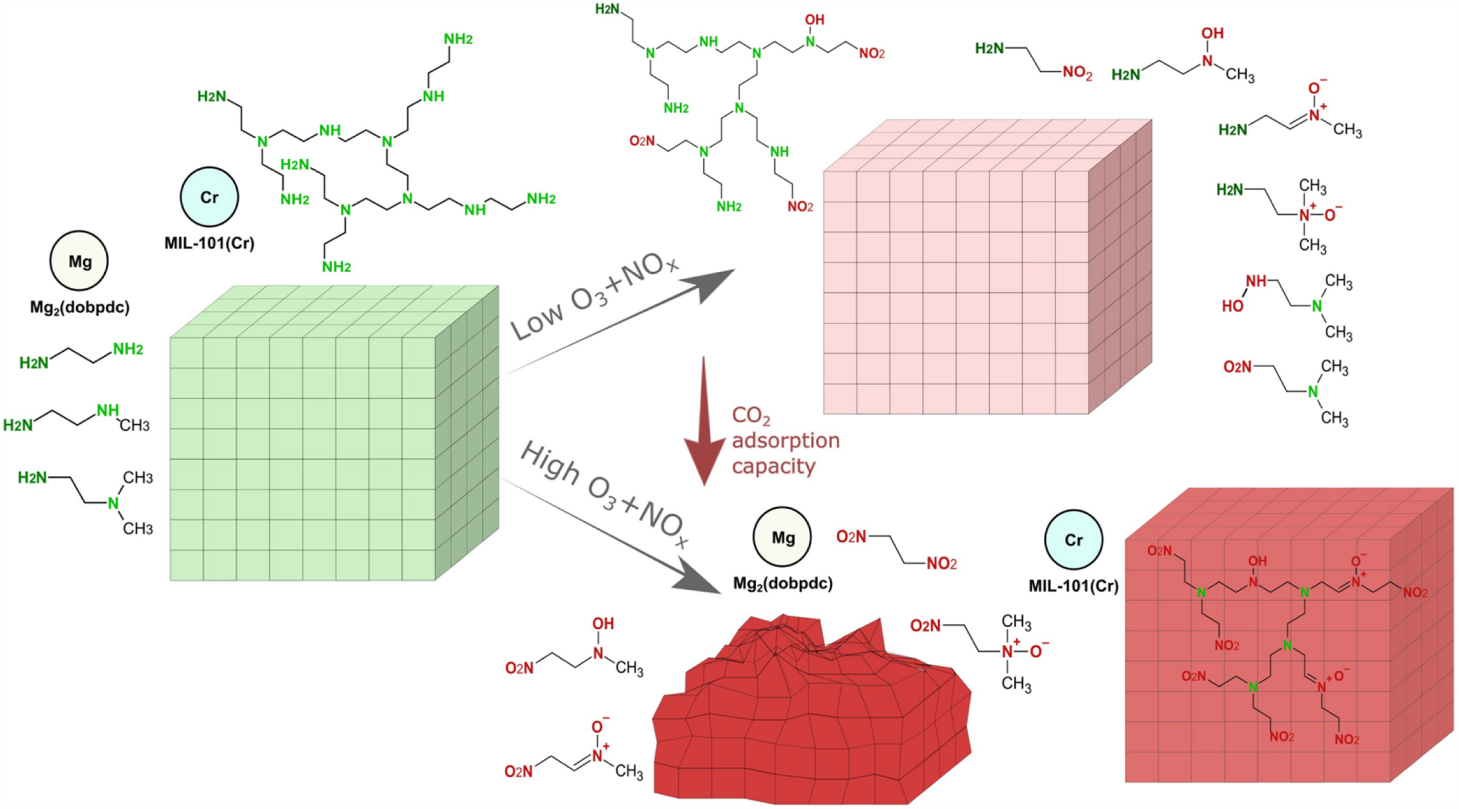
Visual representation of the interaction between ozone and NOx with MOF-amine composites studied in this work.

In industrial-scale DAC processes, CO_2_ is present along with H_2_O. Water is expected to decrease the rate of reaction between ozone and amine groups through the formation of hydrogen bonds that increase the energetic barrier for hydrogen abstraction, as described by Jamdade et al.^15^ CO_2_ would bind to the amines as a Lewis acid, pulling electron density from these functionalities, increasing the lability of their hydrogen groups, and only adding a barrier in terms of steric hindrance in a direct binding mechanism scenario; thus, it would be expected to promote ozone degradation of terminal primary amines. On the other hand, if CO_2_ is adsorbed cooperatively between a primary amine and a Mg metal site before ozone interacts with it, it is likely that it would hinder its degradation; however, it may promote hydrogen abstraction from the terminal-bound amine on the other end of the grafted molecule by pulling electron density from it.

## Conclusions

Grafted amines provide some protection against degradation of the crystalline structure of Mg_2_(dobpdc); however, ozone is more prone to interacting with primary amines rather than with secondary or tertiary amines. Correspondingly, it is expected that primary amines will function as sacrificial functionalities by forming nitro groups after being oxidized by O_3_.

Diamine-appended sorbents like Mg_2_(dobpdc)-en that rely more on direct interaction between CO_2_ and primary amines as their main mechanism of adsorption outperform Mg_2_(dobpdc)-men in structural stability that relies on cooperative interaction with the metal sites of the MOF, as seen from changes in porosity as well as in their diffraction patterns after exposure to ozone.

The mechanism of adsorption dictates how degradation of the material is observed. There is a decrease in step pressure for Mg_2_(dobpdc)-men and Mg_2_(dobpdc)-dmen composites that interact through cooperative adsorption and a direct decrease for Mg_2_(dobpdc)-en and MIL-101­(Cr)-PEI that rely on direct interaction between primary amines and CO_2_. Furthermore, when supported amines do not possess terminal amines that can react directly with oxidizing species such as O_3_, interaction with the metal-bound amine or the support is more likely to occur, as observed for Mg_2_(dobpdc)-dmen even when exposed to 50 ppm of O_3_.

This work has several key limitations. First, the interaction between the sorbents was not assessed by separating the effect of O_3_ and NOx, as ozone was generated directly from ultrazero-grade air, but it was assumed that the concentration of O_3_ in the stream would be higher based on results from the literature. Second, the OX-B431 is cross-sensitive to NO_2_, and an additional electrochemical sensor can be used for removing its influence. However, the NO_2_ sensor NO2-B43F from Alphasense Ltd. possesses an ozone filter with a short lifetime that would have made its measurements vary significantly after a single exposure, and it is also cross-sensitive to NO and N_2_O. Third, the accelerated degradation studies performed in this work are an approximation of the effect that exposure to ground-level ozone and NOx can generate over the course of 1 year, and more work should be performed for modeling purposes, including variables such as thermal cycling as well as the presence of CO_2_ and humidity to represent actual operating conditions. Fourth, this work does not address the effect of temperature on the interaction between ozone and the MOF-amine sorbents. Using air for ozone generation also produces NOx impurities, and the presence of such impurities could increase significantly if the decomposition of ozone is favored by performing the exposures at higher temperatures. Finally, degradation products were not quantified at the outlet of the setup during the exposures or during thermal regeneration, which would allow us to confirm the results observed in the CHN elemental analyses as well as to better understand the environmental implications of these degradation processes as well as rank the reactivity of the compounds to ozone and NOx.

## Supplementary Material



## Notes

Additional material synthesis details, exposure procedure details, adsorption isotherms, ATR-FTIR results, XPS results and peak model references, elemental and thermogravimetric analyses (PDF)

## References

[1] Trickett C. A., Helal A., Al-Maythalony B. A., Yamani Z. H., Cordova K. E., Yaghi O. M. (2017). The Chemistry of Metal–Organic Frameworks for
CO2 Capture, Regeneration and Conversion. Nat.
Rev. Mater..

[2] Bose S., Sengupta D., Rayder T. M., Wang X., Kirlikovali K. O., Sekizkardes A. K., Islamoglu T., Farha O. K. (2024). Challenges and Opportunities:
Metal–Organic Frameworks for Direct Air Capture. Adv. Funct. Mater..

[3] Kalaj M., Cohen S. M. (2020). Postsynthetic Modification:
An Enabling Technology
for the Advancement of Metal–Organic Frameworks. ACS Cent. Sci..

[4] Hu Y., Verdegaal W. M., Yu S.-H., Jiang H.-L. (2014). Alkylamine-Tethered
Stable Metal–Organic Framework for CO2 Capture from Flue Gas. ChemSusChem.

[5] Sriram A., Choi S., Yu X., Brabson L. M., Das A., Ulissi Z., Uyttendaele M., Medford A. J., Sholl D. S. (2024). The Open
DAC 2023 Dataset and Challenges for Sorbent Discovery in Direct Air
Capture. ACS Cent. Sci..

[6] Azarabadi H., Lackner K. S. (2019). A Sorbent-Focused
Techno-Economic Analysis of Direct
Air Capture. Appl. Energy.

[7] Holmes H. E., Banerjee S., Vallace A., Lively R. P., Jones C. W., Realff M. J. (2024). Tuning Sorbent Properties
to Reduce the Cost of Direct
Air Capture. Energy Environ. Sci..

[8] Hunt R., Gillbanks J., Czapla J., Wan Z., Karmelich C., White C., Wood C. (2024). Representative Longevity Testing
of Direct Air Capture Materials. Chem. Eng.
J..

[9] Guta Y. A., Carneiro J., Li S., Innocenti G., Pang S. H., Sakwa-Novak M. A., Sievers C., Jones C. W. (2023). Contributions
of CO2, O2, and H2O to the Oxidative Stability of Solid Amine Direct
Air Capture Sorbents at Intermediate Temperature. ACS Appl. Mater. Interfaces.

[10] Drage T. C., Arenillas A., Smith K. M., Snape C. E. (2008). Thermal Stability
of Polyethylenimine Based Carbon Dioxide Adsorbents and Its Influence
on Selection of Regeneration Strategies. Microporous
Mesoporous Mater..

[11] Khatri R. A., Chuang S. S. C., Soong Y., Gray M. (2006). Thermal and Chemical
Stability of Regenerable Solid Amine Sorbent for CO2 Capture. Energy Fuels.

[12] Pyo S. W., Manianglung C., Ko Y. S. (2025). In-Situ IR Study on Stability of
Epoxide-Functionalized Polyethyleneimine CO2 Adsorbent to NO2 and
SO2 Gases. Korean J. Chem. Eng..

[13] Lewis P. M. (1986). Effect
of Ozone on Rubbers: Countermeasures and Unsolved Problems. Polym. Degrad. Stab..

[14] Iwase Y., Shindo T., Kondo H., Ohtake Y., Kawahara S. (2017). Ozone Degradation
of Vulcanized Isoprene Rubber as a Function of Humidity. Polym. Degrad. Stab..

[15] Jamdade S., Cai X., Sholl D. S. (2025). Assessment
of Long-Term Degradation of Adsorbents for
Direct Air Capture by Ozonolysis. J. Phys. Chem.
C.

[16] Yue C., Wu L., Lin Y., Lu Y., Shang C., Ma R., Zhang X., Wang X., Wu W. D., Chen X. D., Wu Z. (2021). Study on the Stability, Evolution of Physicochemical Properties,
and Postsynthesis of Metal–Organic Frameworks in Bubbled Aqueous
Ozone Solution. ACS Appl. Mater. Interfaces.

[17] Dong C., Yang J.-J., Xie L.-H., Cui G., Fang W.-H., Li J.-R. (2022). Catalytic Ozone Decomposition and Adsorptive VOCs Removal in Bimetallic
Metal-Organic Frameworks. Nat. Commun..

[18] Cramariuc, R. ; Velisar, I. ; Milevschi, V. ; Munteanu, V. ; Ghiuta, V. ; Tanasescu, F. T. New Considerations of Ozone Generation and the Influence of NOx in Ozone Production and Water Treatment. In The Modern Problems of Electrostatics with Applications in Environment Protection, Inculet, I. I. ; Tanasescu, F. T. ; Cramariuc, R. eds.; Springer, Dordrecht, 1999; pp. 313–340. 10.1007/978-94-011-4447-6_21.

[19] Pontiga1, F. ; Castellanos, A. Nitrogen Oxides Generation Induced by Negative Corona Discharge in N2 + 02 Mixtures. In 2006 IEEE Conference on Electrical Insulation and Dielectric Phenomena; IEEE: 2006, pp. 264–267. DOI: 10.1109/CEIDP.2006.312112

[20] Darunte L. A., Terada Y., Murdock C. R., Walton K. S., Sholl D. S., Jones C. W. (2017). Monolith-Supported Amine-Functionalized Mg2­(Dobpdc)
Adsorbents for CO2 Capture. ACS Appl. Mater.
Interfaces.

[21] Bose S., Sengupta D., Malliakas C. D., Idrees K. B., Xie H., Wang X., Barsoum M. L., Barker N. M., Dravid V. P., Islamoglu T., Farha O. K. (2023). Suitability of a Diamine Functionalized
Metal–Organic Framework for Direct Air Capture. Chem. Sci..

[22] Song M., Rim G., Mirzazadeh G., Hoffman J., Moon H. J., Leisen J. E., Nik O. G., Lively R. P., Jones C. W. (2026). Amine-Dependent
CO 2 Sorption on Amine-Impregnated Mg 2 (dobpdc) MOF under Humid Conditions. Ind. Chem. Mater..

[23] Holmes H. E., Ghosh S., Li C., Kalyanaraman J., Realff M. J., Weston S. C., Lively R. P. (2023). Optimum
Relative
Humidity Enhances CO2 Uptake in Diamine-Appended M2­(Dobpdc). Chem. Eng. J..

[24] Darunte L. A., Oetomo A. D., Walton K. S., Sholl D. S., Jones C. W. (2016). Direct
Air Capture of CO2 Using Amine Functionalized MIL-101­(Cr). ACS Sustainable Chem. Eng..

[25] Jiang K., Yang J., Liu X., Tong Y., Liu J., Gu J. (2025). Green Synthesis of
MIL-101­(Cr) with Enhanced Direct Air Capture of
CO2 through Synergistic Effects of Polyethyleneimine and Additives. Chem. Eng. J..

[26] Lin Y., Yan Q., Kong C., Chen L. (2013). Polyethyleneimine Incorporated Metal-Organic
Frameworks Adsorbent for Highly Selective CO2 Capture. Sci. Rep..

[27] Llewellyn P. L., Bourrelly S., Serre C., Vimont A., Daturi M., Hamon L., De Weireld G., Chang J.-S., Hong D.-Y., Kyu Hwang Y., Hwa Jhung S., Férey G. (2008). High Uptakes
of CO2 and CH4 in Mesoporous Metal—Organic Frameworks MIL-100
and MIL-101. Langmuir.

[28] Sheikh
Alivand M., Hossein Tehrani N. H.
M., Shafiei-Alavijeh M., Rashidi A., Kooti M., Pourreza A., Fakhraie S. (2019). Synthesis
of a Modified HF-Free MIL-101­(Cr) Nanoadsorbent with Enhanced H2S/CH4,
CO2/CH4, and CO2/N2 Selectivity. J. Environ.
Chem. Eng..

[29] Siegelman R. L., McDonald T. M., Gonzalez M. I., Martell J. D., Milner P. J., Mason J. A., Berger A. H., Bhown A. S., Long J. R. (2017). Controlling
Cooperative CO2 Adsorption in Diamine-Appended Mg2­(Dobpdc) Metal–Organic
Frameworks. J. Am. Chem. Soc..

[30] Vitillo J. G., Bordiga S. (2017). Increasing the Stability of Mg2­(Dobpdc) Metal–Organic
Framework in Air through Solvent Removal. Mater.
Chem. Front..

[31] Parker S. T., Smith A., Forse A. C., Liao W.-C., Brown-Altvater F., Siegelman R. L., Kim E. J., Zill N. A., Zhang W., Neaton J. B., Reimer J. A., Long J. R. (2022). Evaluation of the
Stability of Diamine-Appended Mg2­(Dobpdc) Frameworks to Sulfur Dioxide. J. Am. Chem. Soc..

[32] Min Y. J., Ganesan A., Realff M. J., Jones C. W. (2022). Direct Air Capture
of CO2 Using Poly­(Ethyleneimine)-Functionalized Expanded Poly­(Tetrafluoroethylene)/Silica
Composite Structured Sorbents. ACS Appl. Mater.
Interfaces.

[33] Li C., Wang X., Yang A., Chen P., Zhao T., Liu F. (2021). Polyethyleneimine-Modified Amorphous Silica for the Selective Adsorption
of CO2/N2 at High Temperatures. ACS Omega.

[34] Environmental Protection Agency National Ambient Air Quality Standards For Ozone; Federal Register. 2015. https://www.federalregister.gov/documents/2015/10/26/2015-26594/national-ambient-air-quality-standards-for-ozone.

[35] Karlsson P. E., Klingberg J., Engardt M., Andersson C., Langner J., Karlsson G. P., Pleijel H. P. (2017). Present and Future
Concentrations of Ground-Level Ozone and Potential Impacts on Ecosystems
and Human Health in Northern Europe. Sci. Total
Environ..

[36] Hadji K., Pontiga F., Belasri A., Hadj-Ziane S., Fernández-Rueda A. (2014). Experimental Study of Ozone Generation
by Negative Corona Discharge in Mixtures of N2 and O2. Ozone: Sci. Eng..

[37] Lim S., McArdell C. S., von Gunten U. (2019). Reactions
of Aliphatic Amines with
Ozone: Kinetics and Mechanisms. Water Res..

[38] Fine N. A., Rochelle G. T. (2014). Absorption of Nitrogen
Oxides in Aqueous Amines. Energy Procedia.

[39] Reed B. P., Marchesini S., Chemello G., Morgan D. J., Vyas N., Howe T., Radnik J., Clifford C. A., Pollard A. J. (2023). The Influence
of Sample Preparation on XPS Quantification of Oxygen-Functionalised
Graphene Nanoplatelets. Carbon.

[40] Scofield J. H. (1976). Hartree-Slater
Subshell Photoionization Cross-Sections at 1254 and 1487 eV. J. Electron Spectrosc. Relat. Phenom..

[41] Seah M. P. (2012). Simple
Universal Curve for the Energy-Dependent Electron Attenuation Length
for All Materials. Surf. Interface Anal..

[42] Fairley N., Fernandez V., Richard-Plouet M., Guillot-Deudon C., Walton J., Smith E., Flahaut D., Greiner M., Biesinger M., Tougaard S., Morgan D., Baltrusaitis J. (2021). Systematic
and Collaborative Approach to Problem Solving Using X-Ray Photoelectron
Spectroscopy. Appl. Surf. Sci. Adv..

[43] Shirley D. A. (1972). High-Resolution
X-Ray Photoemission Spectrum of the Valence Bands of Gold. Phys. Rev. B.

[44] Osterrieth J. W. M., Rampersad J., Madden D., Rampal N., Skoric L., Connolly B., Allendorf M. D., Stavila V., Snider J. L., Ameloot R., Marreiros J., Ania C., Azevedo D., Vilarrasa-Garcia E., Santos B. F., Bu X.-H., Chang Z., Bunzen H., Champness N. R., Griffin S. L., Chen B., Lin R.-B., Coasne B., Cohen S., Moreton J. C., Colón Y. J., Chen L., Clowes R., Coudert F.-X., Cui Y., Hou B., D’Alessandro D.
M., Doheny P. W., Dincă M., Sun C., Doonan C., Huxley M. T., Evans J. D., Falcaro P., Ricco R., Farha O., Idrees K. B., Islamoglu T., Feng P., Yang H., Forgan R. S., Bara D., Furukawa S., Sanchez E., Gascon J., Telalović S., Ghosh S. K., Mukherjee S., Hill M. R., Sadiq M. M., Horcajada P., Salcedo-Abraira P., Kaneko K., Kukobat R., Kenvin J., Keskin S., Kitagawa S., Otake K., Lively R. P., DeWitt S. J. A., Llewellyn P., Lotsch B. V., Emmerling S. T., Pütz A. M., Martí-Gastaldo C., Padial N. M., García-Martínez J., Linares N., Maspoch D., Suárez Del Pino J. A., Moghadam P., Oktavian R., Morris R. E., Wheatley P. S., Navarro J., Petit C., Danaci D., Rosseinsky M. J., Katsoulidis A. P., Schröder M., Han X., Yang S., Serre C., Mouchaham G., Sholl D. S., Thyagarajan R., Siderius D., Snurr R. Q., Goncalves R. B., Telfer S., Lee S. J., Ting V. P., Rowlandson J. L., Uemura T., Iiyuka T., van der Veen M. A., Rega D., Van Speybroeck V., Rogge S. M. J., Lamaire A., Walton K. S., Bingel L. W., Wuttke S., Andreo J., Yaghi O., Zhang B., Yavuz C. T., Nguyen T. S., Zamora F., Montoro C., Zhou H., Kirchon A., Fairen-Jimenez D. (2022). How Reproducible Are Surface Areas Calculated from
the BET Equation?. Adv. Mater..

[45] Sapnik F., Johnstone D. N., Collins S. M., Divitini G., Bumstead A. M., Ashling C. W., Chater P. A., Keeble D. S., Johnson T., Keen A. (2021). Stepwise collapse
of a giant pore metal–organic framework. Dalton Trans..

[46] Bhattacharyya S., Han R., Joshi J. N., Zhu G., Lively R. P., Walton K. S., Sholl D. S., Nair S. (2019). Stability of Zeolitic Imidazolate
Frameworks in NO2. J. Phys. Chem. C.

[47] Rezaei F., Jones C. W. (2013). Stability of Supported Amine Adsorbents to SO2 and
NOx in Postcombustion CO2 Capture. 1. Single-Component Adsorption. Ind. Eng. Chem. Res..

[48] Peeters W., Neerup R., Fosbøl P. L. (2025). Solvent
Degradation & Influences
on Amine-Based Carbon Capture Operations. Int.
J. Greenhouse Gas Control.

[49] Agustika D. K., Mercuriani I., Purnomo C. W., Hartono S., Triyana K., Iliescu D. D., Leeson M. S. (2022). Fourier Transform Infrared Spectrum
Pre-Processing Technique Selection for Detecting PYLCV-Infected Chilli
Plants. Spectrochim. Acta, A.

[50] Badertscher, M. ; Bühlmann, P. ; Pretsch, E. Structure Determination of Organic Compounds; Springer: Berlin, Heidelberg, 2009. DOI: 10.1007/978-3-540-93810-1.

[51] Fahlman A., Hamrin K., Hedman J., Nordberg R., Nordling C., Siegbahn K. (1966). Electron Spectroscopy
and Chemical Binding. Nature.

[52] Leblond L., Anagri A., Fiset J., Borget M.-Y., Bébin P., Dumais N., Vuillaume Y. (2024). Polypropylene
Fabric Coated with
Branched Polyethyleneimine Derivatives for High Antiviral Activity. RSC Applied Interfaces.

[53] Ardizzone S., Bianchi C. L., Fadoni M., Vercelli B. (1997). Magnesium Salts and
Oxide: An XPS Overview. Appl. Surf. Sci..

