# Supplementary material for: Stability of Metal–Organic Framework-Supported Amines under Exposure to Ozone Generated from Air
Source: Ind Eng Chem Res. 2026 Jul 9;65(28):14930–40. doi: 10.1021/acs.iecr.6c01324 (PMC13397569; doi:10.1021/acs.iecr.6c01324)
Supplement: Supplementary file 1 [file ie6c01324_si_001.pdf]

Supporting Information

Stability of metal-organic framework-supported amines under exposure to ozone generated from air

Mario Zorrilla Valtierra[†], Botagoz Kuspangaliyeva[†], Yuhe Cao[†], Jonas Baltrusaitis[‡], Ryan P. Lively[†]*, Krista S. Walton[†§]*

[†] School of Chemical & Biomolecular Engineering, Georgia Institute of Technology, 311 Ferst Drive, Atlanta, Georgia 30332, United States

[‡]Department of Chemical and Biomolecular Engineering, Lehigh University, 111 Research Dr., Bethlehem, PA 18015, USA

[§]Department of Chemical and Biomolecular Engineering, North Carolina State University, 911 Partners Way, Raleigh NC 27695, USA

*email: ryan.lively@chbe.gatech.edu; kswalton@ncsu.edu

Table of Contents

MIL-101(Cr) washing procedure with NH_4OH 3

Test for stability of Mg_2(dobpdc) 5

Equipment and exposure unit setup design 5

Preliminary tests with ozone exposure equipment 6

X-ray photoelectron spectroscopy sample preparation 8

Color changes during exposure to ozone 8

Second set of experiments (full ozone generation capacity and undiluted air) 10

Nitrogen isotherms at 77 K for porosimetry 15

CO_2 adsorption isotherms at 298 K 17

CO_2 dynamic adsorption measurements with 400 ppm carbon dioxide in N_2 at 298 K 19

Additional powder diffraction patterns 19

ATR-FTIR spectra of composites before and after exposure 20

X-ray photoelectron core spectra 25

Elemental analysis flask combustion CHN 33

C 1s 285 eV referencing 34

Thermogravimetric analysis 35

References 35

MIL-101(Cr) washing procedure with NH_4OH

After centrifuging the tubes and disposing of the mother solution, the samples were refilled with DI water, left for at least 3 hr at 50 °C in a sonicating bath and centrifuged at 5800 rpm. This washing procedure with water was repeated three times followed by an overnight water wash under the same conditions. Following this, the sample was washed in a sonicating bath at 50 °C for at least 12 hr per iteration alternating between one iteration by washing with 50mM aqueous NH_4OH solution and two iterations by washing with deionized water, centrifuging and disposing of the supernatant liquid after each wash. This NH_4OH-water washing process was repeated once more, but the time for the water soaking iterations was modified to 3 hr followed by a final water soak for at least 12 hr at 50°C while sonicating. Consecutively, a solvent-exchange with methanol was performed by doing three iterations of soaking the sample in fresh methanol at 50 °C while sonicating overnight.

The procedure for washing with DMF used for comparison of the ATR-FTIR spectra shown in Figures S1 and S2 was similar to that followed by Darunte et. al.[1]. In brief, 3 iterations of soaking in DMF are subsequent to 3 iterations of soaking methanol, and the solvent-exchange procedure between DMF and methanol is the previously discussed.

The washing process implemented in this work differs from some of the literature procedures[1–3] since the first washes are performed with water to remove any of the unreacted chromium salt and N,N-dimethylformamide (DMF) is replaced with a 50 mM aqueous solution of NH_4OH inspired from the procedure followed by Llewellyn et. al. [4,5]) that consists of the use of NH_4F in an attempt to remove as much BDC as possible from the framework. Removing as much BDC as possible prevents the FTIR absorbance bands associated with free BDC found at approximately 1288 cm^{-1} from overlapping with expected products from amine oxidation that can be found within the same vibrational frequency range (i.e. N-O stretching bands at around 1300 cm^{-1}) as shown in Figures S1-S2. It would seem as if washing MIL-101(Cr) with NH4OH does not have a significant effect on MIL-101(Cr)-PEI, but the strong band between 1250-1500 cm^{-1} is better defined for the PEI impregnated sample that was washed with NH_4OH as well as some subtle differences in the intensities of bands at approximately 810 cm^{-1}

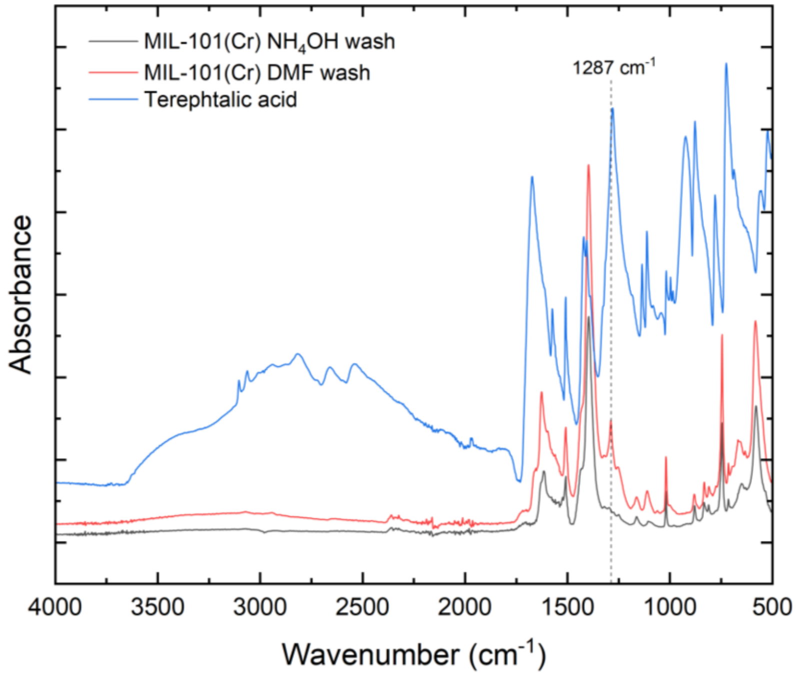

Figure S1. Comparison between ATR-FTIR spectra of MIL-101(Cr) washed with DMF (red) and with NH_4OH solution (black). Spectra of H_2BDC included for comparison purposes (blue)

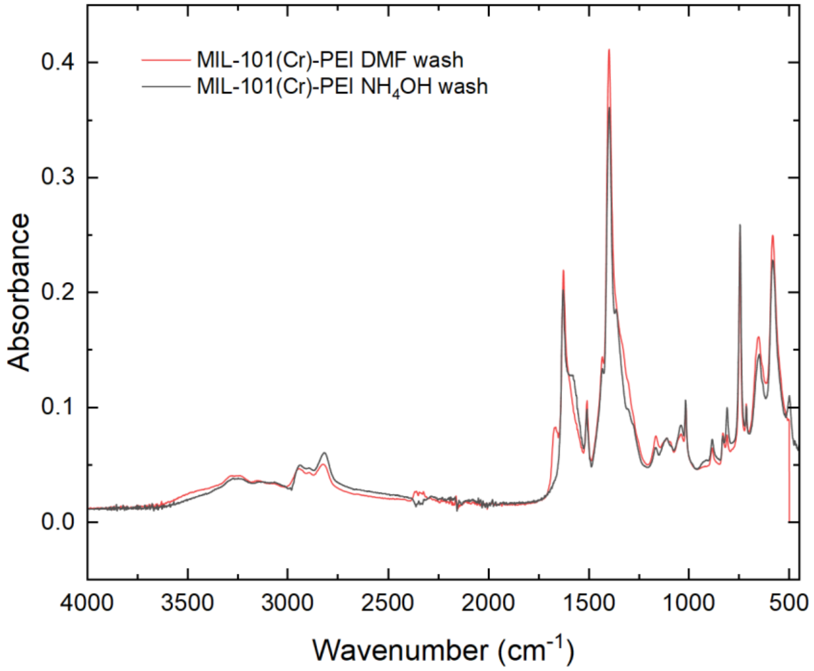

Figure S2. Comparison between ATR-FTIR spectra of MIL-101(Cr)-PEI washed with DMF (red) and washed with NH_4OH (black).

Test for stability of Mg_2(dobpdc)

The surface area and pore volume of Mg_2(dobpdc) samples was obtained from N_2 isotherms performed at 77 K using a Micromeritics Tristar II Plus after synthesis through activation at 180 °C for 16 hr subsequent to being submitted to desolvation at the same conditions inside a vacuum oven. The sample recipient used for loading aliquots of the samples to the adsorption tubes were left under vacuum overnight and backfilled with N_2 using a vacuum oven. These samples were analyzed after a month of being kept inside a vacuum desiccator. The N_2 porosimetry results from the sample synthesized for performing preliminary tests as well as the batch that was used for exposure to low concentration of ozone are shown on Figure S3.

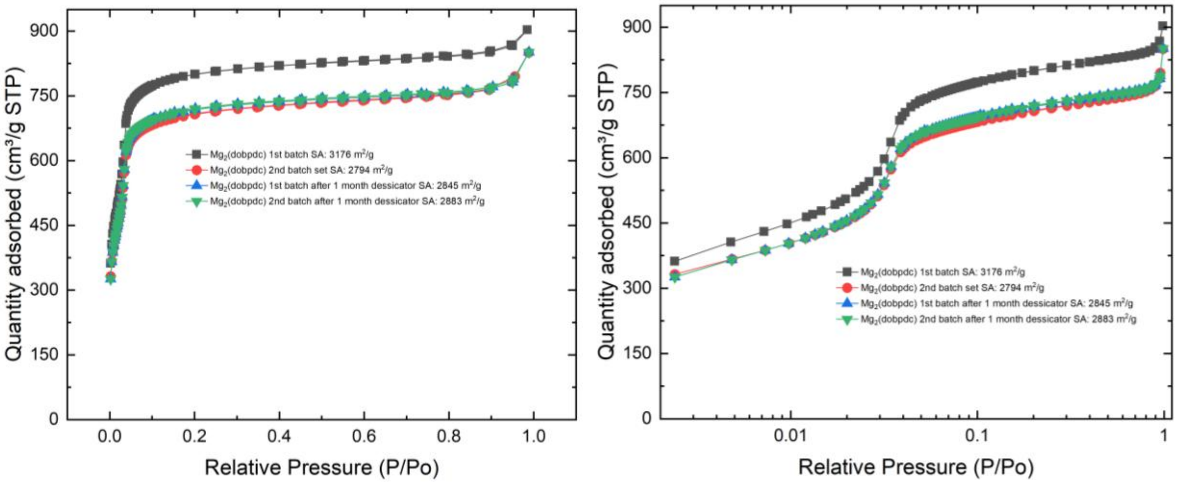

Figure S3. N_2 isotherm for Mg_2(dobpdc) samples as synthesized and after 1 month of being kept inside a vacuum desiccator with their vessels backfilled with N_2. Logarithmic pressure scale isotherm (right).

As shown on Figure S3 there is little loss in surface area and pore volume for the first synthesized batch intended for preliminary ozone exposure tests, while there is no loss of porosity for the second synthesized batch intended for exposure to low concentrations of ozone generated from synthetic air. Thus, it is concluded that degradation from atmospheric humidity is negligible when samples are being kept backfilled with N_2 inside a vacuum desiccator.

Equipment and exposure unit setup design

A commercially available 8 kV ozone generator model MP-8000 acquired from A2Z Ozone Inc, PTFE plastic and stainless-steel tubing as well as stainless fittings and valves were used to build the custom-exposure setup shown in Figure S4. The packed bed consisted of a 6 mm diameter Supelco thermal desorption tube for preliminary tests held by ultra-torr vacuum fittings and was later upsized to a 20 mm glassblown tube with a fine porosity fused silica frit purchased from Chemglass, Inc for the low and high concentration exposure experiments to achieve a more uniform exposure of the sample.

The inlet to the ozone generator was connected directly to an in-line relieving pressure regulator set at 3 psi as specified in the manufacturer manual, which in turn was connected directly to the low-pressure side of a non-relieving gas pressure regulator attached to the ultra-zero grade air tank set. The outlet of the generator was connected to 0-20 sccm stainless steel housing gas mass flow

controller (MFC) purchased from Omega Engineering, which other end was directed to a stainless-steel tee as mixing point first going through a check-valve to prevent backflow from the dilutant line. The other end of the tee was connected to the dilutant line where a 200 sccm gas MFC was used to flow ultra-high purity N_2 to control the concentration of O_3 and decrease the concentration of O_2 as much as possible.

This resulting flow coming from the tee was fed to a stainless-steel tee where one end was connected to an outgassing regulating valve to relieve the excess flow not used after dilution, and the other end was connected to a 0-100 sccm gas MFC controlling the flow of the oxygen deficient ozone containing air stream that the sample would be exposed to. The outlet from this MFC was connected to a manifold with a three-way valve at the inlet used to control the direction of the flow either through a bypass line or through the packed bed loaded with the sample. The outlet of the packed bed side and the bypass side were connected to another three-way valve that would separate both and select which of the sides to be directly connected to the sensor array.

The sensor array consisted of three pre-made and commercially available submersible aluminum enclosures modified with through-wall stainless-steel fittings and adapted to hold the sensors upside-down as close as possible to the level of the flow. Each enclosure would house either an ozone electrochemical sensor model OX-B431 (0-20 ppm O_3) with a resolution of 0.1 ppm or an oxygen A2-O2 (0-30%) electrochemical sensor acquired from Alphasense Inc. It is worth noting that the ozone sensor OX-B431 maximum value is 20 ppm; however, it can measure up to 50 ppm according to the supplier even though this condition shortens its lifetime. To achieve this concentration, 13 sccm of ultra zero grade air were feed into the generator at 50% of its ozone generation capacity that were further diluted with 180 sccm of UHP N_2. A portion of this resulting stream is vented, while 50 sccm of the mix can either be fed to the fixed bed, or be directed through the bypass towards the sensors.

The ozone exposure setup was safely located within a fume hood to avoid exposure to hazardous levels of ozone and a BW Solo O_3 Honeywell safety sensor (0-1 ppm) was used as a precaution in case of ozone leaking into the room.

Preliminary tests with ozone exposure equipment

The objective of this preliminary test is to provide evidence of the impact of ground-level ozone on the chemical structure of metal-organic frameworks (MOFs) supporting amine compounds. Two composite materials composed by Mg_2(dobpdc) appended with one of the two different isomers of N,N'-dimethylethylenediamine (dmen) are exposed to a stream of oxygen-deficient air containing 40 ppm of O_3 generated from synthetic air using the custom-made experimental setup shown on Figure S4 with the difference that these first tests were performed using a 6 mm diameter glass tube that was changed for a 20 mm tube for the future experiments to achieve a more homogeneous interaction between O_3 and the sample. The exposures were maintained for approximately 30 min to simulate a time weighed average of 1 ppm-day exposure. A concentration profile for the experiment as well as a picture of the sample after exposure are shown on Figure S5.

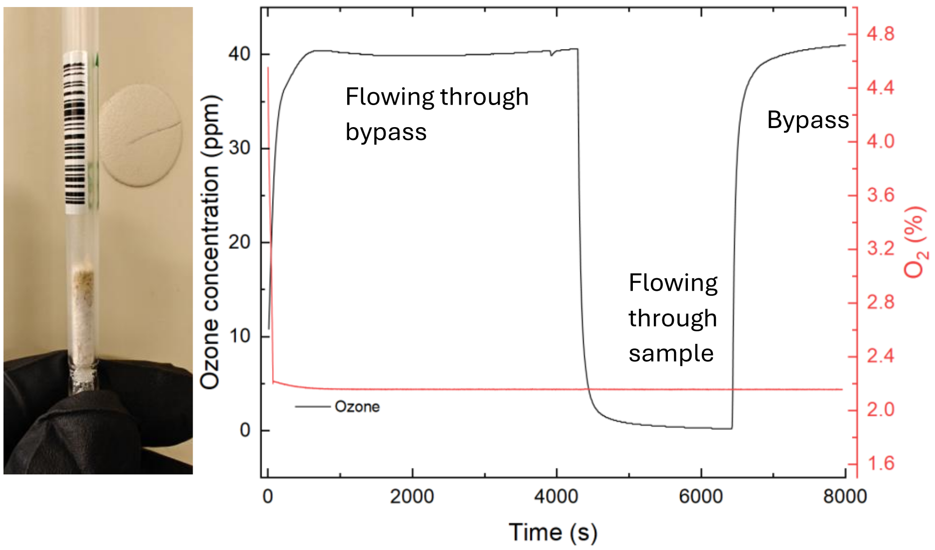

Figure S4. Mg2(dobpdc)-dmen sample after 30 min exposure to a flow of oxygen-defficient air containing 40 ppm O3

ATR-FTIR characterization of the amine solid sorbents shows changes due to the presence of water (3000-3500 cm^{-1}) and potentially nitro moieties (~1500 cm^{-1} and ~1300 cm^{-1}) after the samples are exposed to O_3 (Figure S6); a change in color is noticeable as well. As a result, the electron-density present on the amine sites of the sorbents is reduced as observed from XPS N(1s) core analysis before and after exposure as a subtle shift in binding energy of the amine peak from 399.47 eV to 399.25 eV while no changes for the N(1s) core analysis of the other isomer are observed (Figure S7).

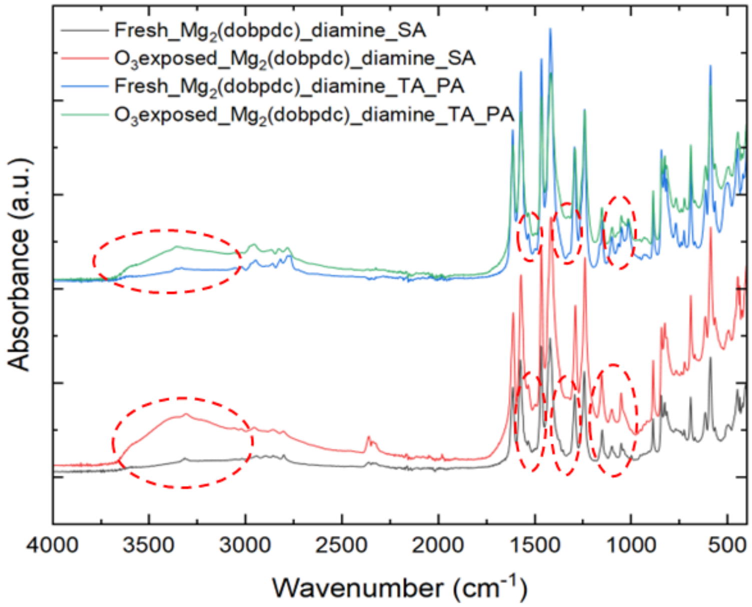

Figure S5. ATR-FTIR spectra of Mg2(dobpdc)-dmen samples with two different isomers appended. SA label refers to isomer with two secondary amines and label TA_PA refers to the sample featuring a primary and a tertiary amine.

X-ray photoelectron spectroscopy sample preparation

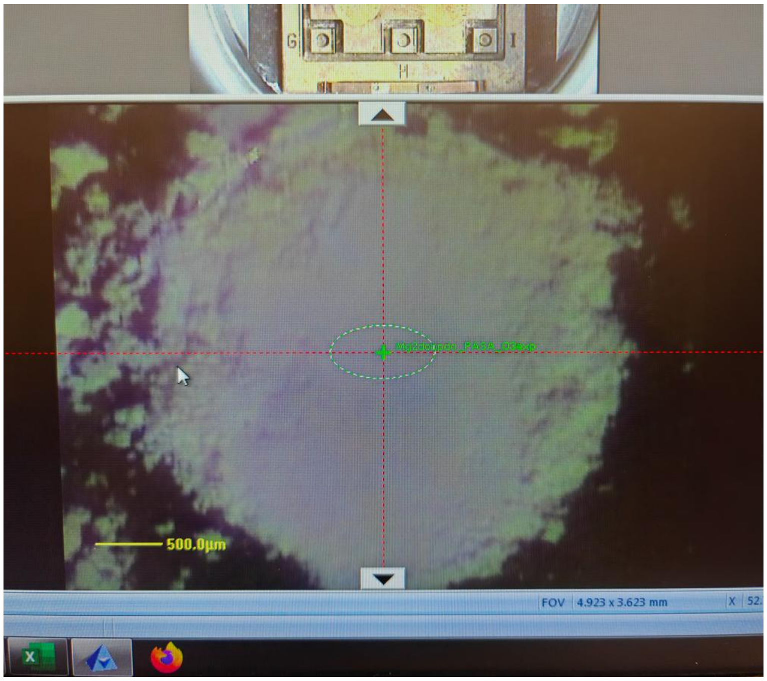

Figure S6. Sample preparation in multiple recess sample holder for XPS analysis

Color changes during exposure to ozone

First set of experiments (50 ppm ozone in oxygen-deficient air)

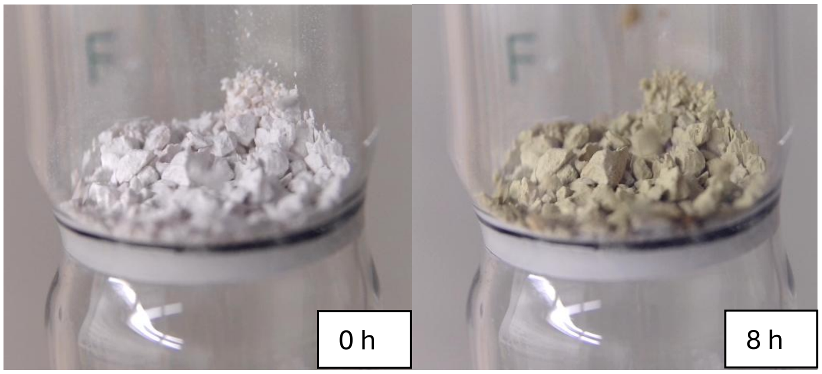

Figure S7. Change in color of Mg_2(dobpdc) during exposure to oxygen-deficient air containing 50 ppm of O_3

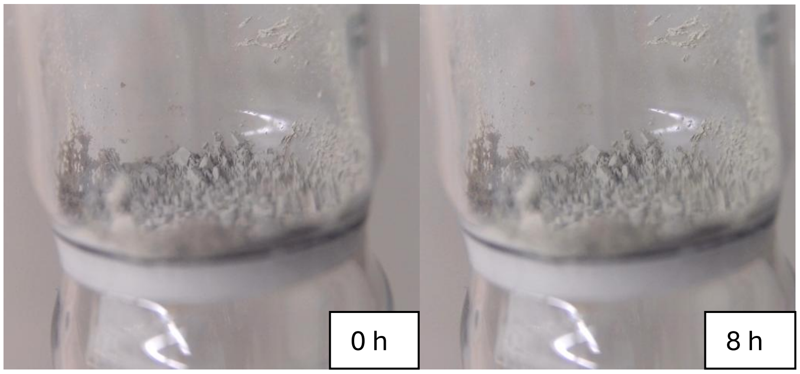

Figure S8. Color of MIL-101(Cr)-PEI before and after exposure to oxygen-deficient air containing 50 ppm of O_3

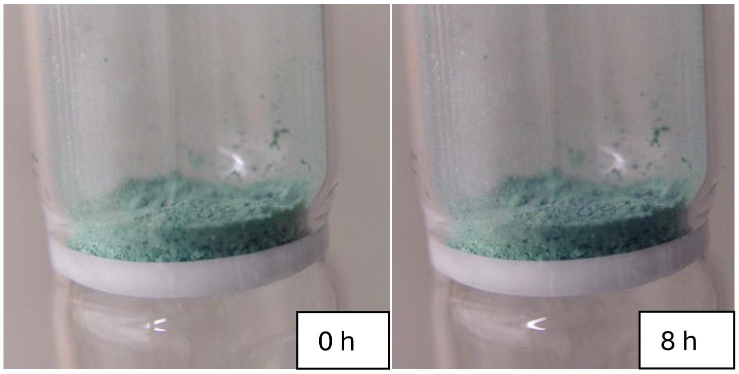

Figure S9. Color of MIL-101(Cr) before and after exposure to oxygen-deficient air containing 50 ppm of O_3

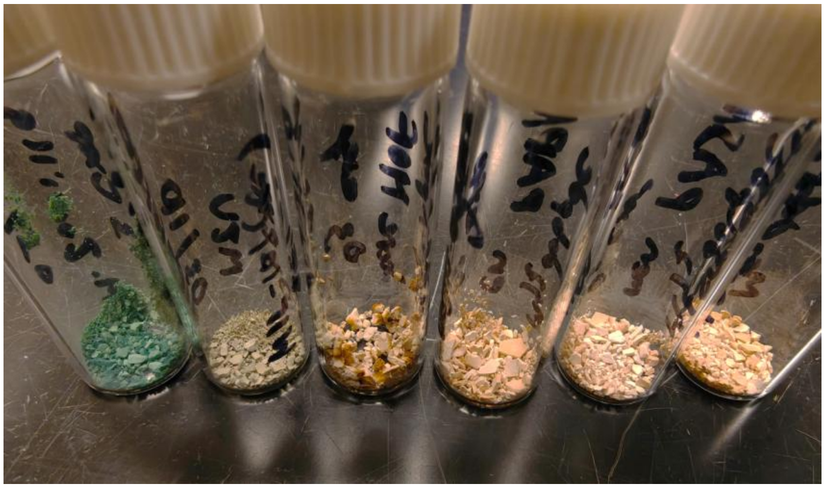

Figure S10. Color of all MOF samples after reactivation after exposure to oxygen-deficient air containing 50 ppm of O_3. From left to right: MIL-101(Cr), MIL-101(Cr)-PEI, Mg_2(dobpdc), Mg_2(dobpdc)-en, Mg_2(dobpdc)-men and Mg_2(dobpdc)-dmen. Lighter and darker sections are observed in all Mg_2(dobpdc) samples that are more evident for Mg_2(dobpdc) bare MOF. Similar color as fresh samples is observed for MIL-101(Cr), but slight more yellow coloration is observed for MIL-101(Cr)-PEI.

Second set of experiments (full ozone generation capacity and undiluted air)

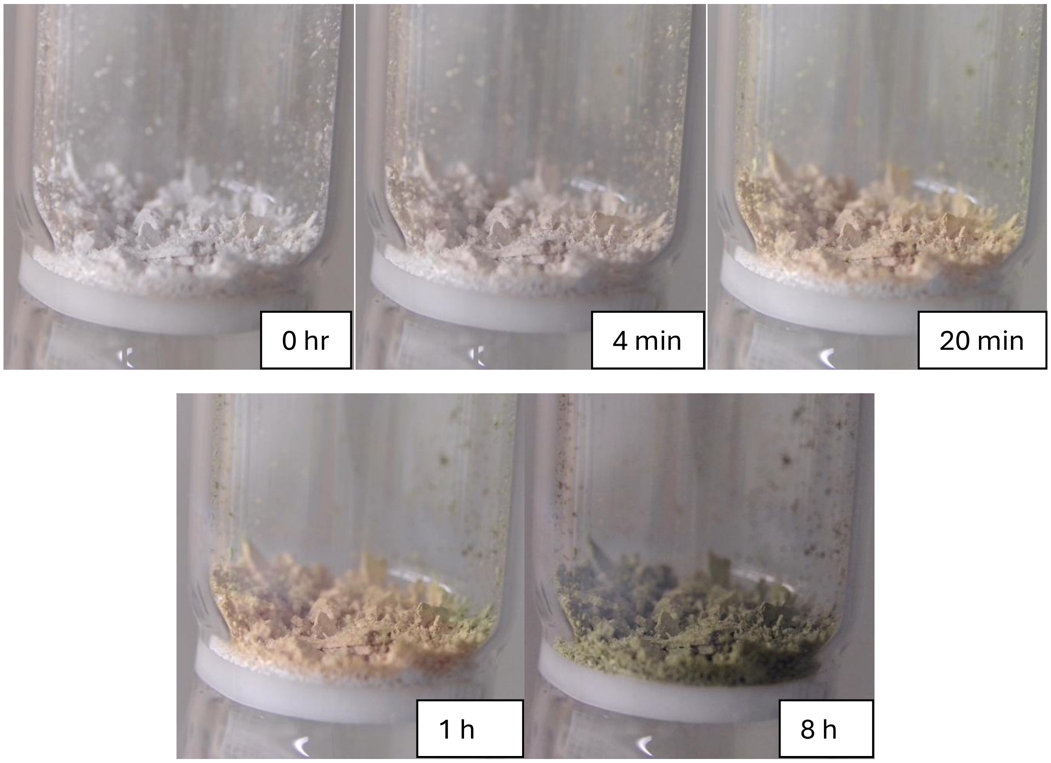

Figure S11. Color changes in Mg_2(dobpdc)-en during exposure to high concentrations of ozone in synthetic air

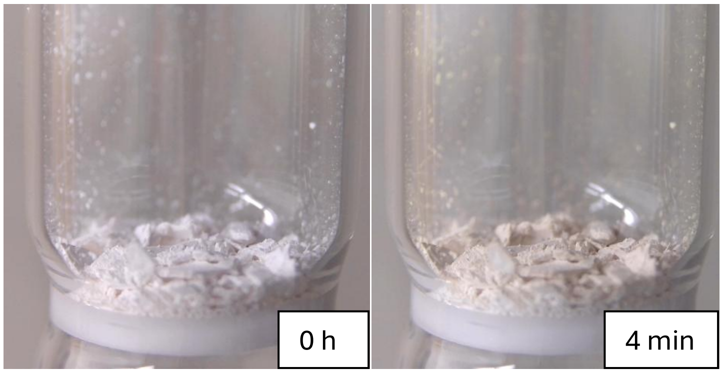

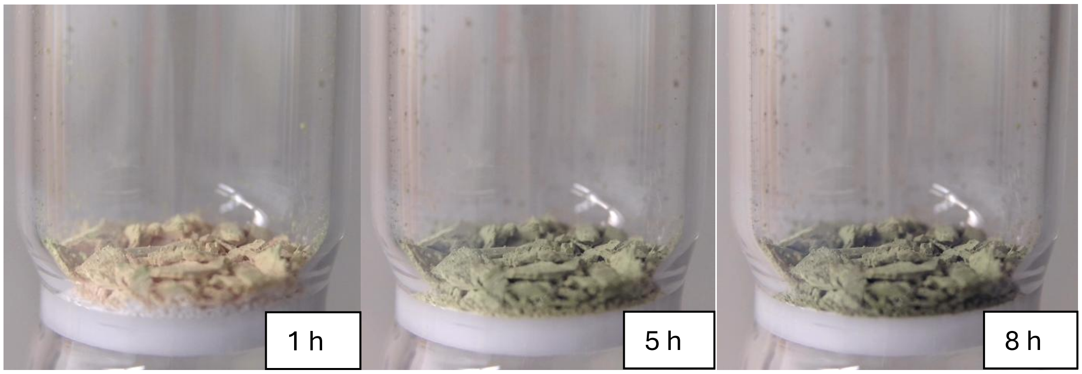

Figure S12. Color changes in Mg2(dobpdc)-men during exposure to high concentrations of ozone in synthetic air

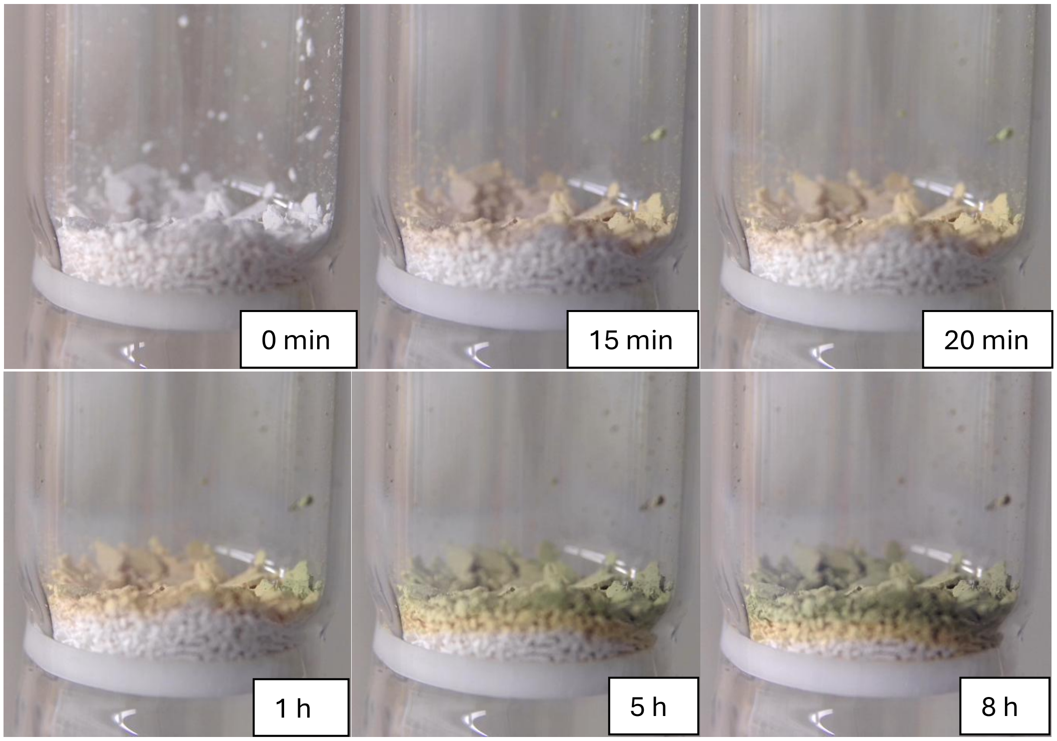

Figure S13. Color changes in Mg2(dobpdc)-dmen during exposure to high concentrations of ozone in synthetic air

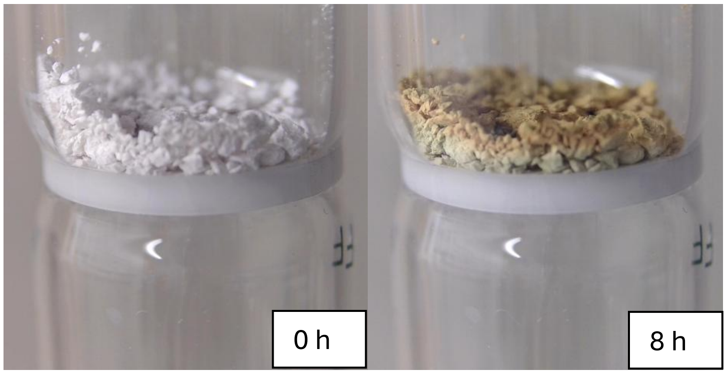

Figure S14. Color changes in Mg2(dobpdc) during exposure to high concentrations of ozone in synthetic air

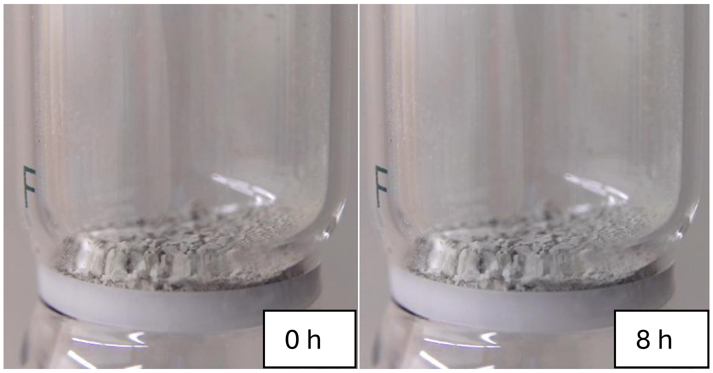

Figure S15. Color changes in MIL-101(Cr)-PEI during exposure to high concentrations of ozone in synthetic air

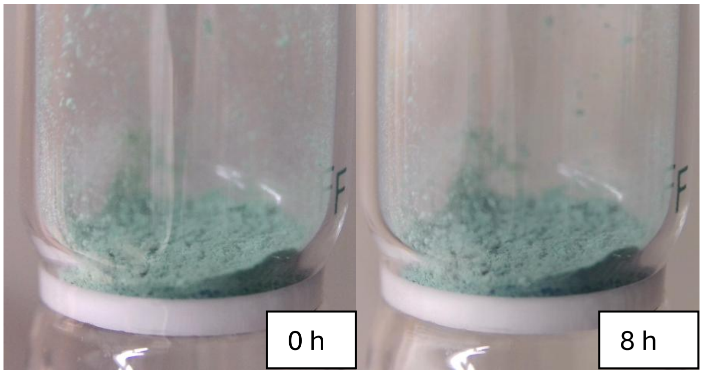

Figure S16. Color changes in MIL-101(Cr) during exposure to high concentrations of ozone in synthetic air

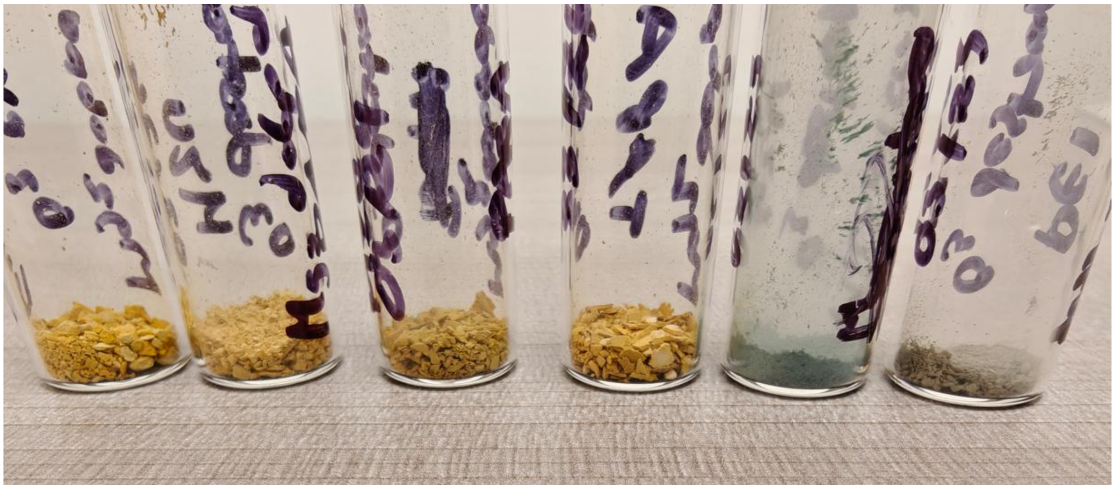

Figure S17. Color of all MOF samples after exposure to air containing high concentrations of O3 and nitrogen oxide byproducts before reactivation. From left to right: MIL-101(Cr), MIL-101(Cr)-PEI, Mg2(dobpdc), Mg2(dobpdc)-en, Mg2(dobpdc)-men and Mg2(dobpdc)-dmen. The green coloration observed in diamine-appended samples during exposure turns into yellowish coloration.

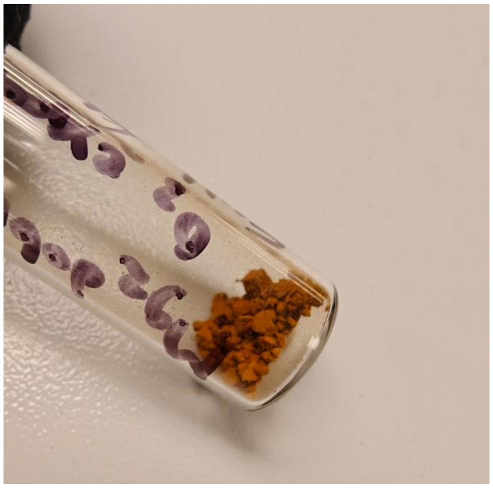

Figure S18. Color of Mg2(dobpdc) after reactivation after exposure to air containing high concentrations of O3 and nitrogen oxide byproducts. Change in the intensity of the yellow color to brownish color suggests the further degradation of the MOF during reactivation.

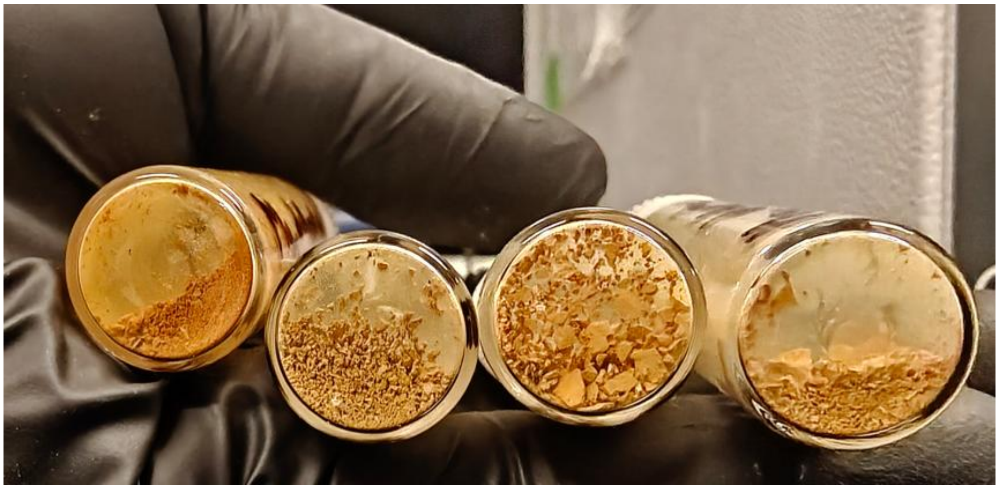

Figure S19. Color of Mg2(dobpdc) MOF and diamine-appended samples after reactivation after exposure to air containing high concentrations of O3 and nitrogen oxide byproducts. From left to right Mg2(dobpdc), Mg2(dobpdc)-en, Mg2(dobpdc)-men, Mg2(dobpdc)-dmen. Darker coloration was observed comparing before and after reactivation.

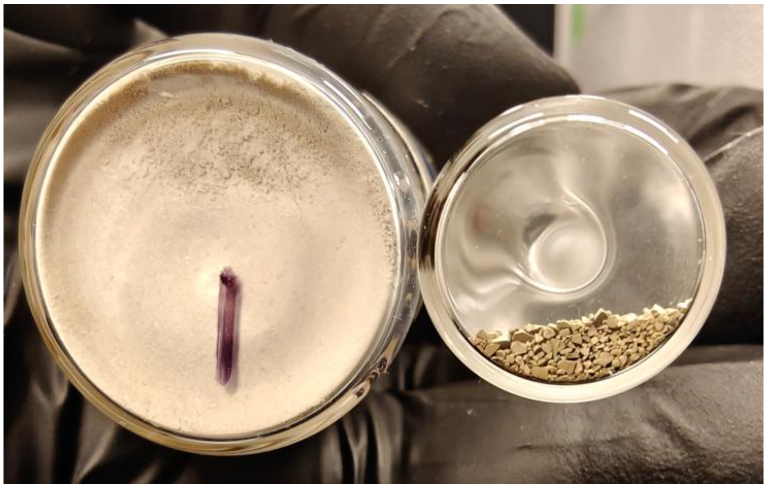

Figure S20. Color comparison MIL-101(Cr)-PEI before (left) and after reactivation after exposure (left) to air containing high concentrations of O3 and nitrogen oxide byproducts.

Nitrogen isotherms at 77 K for porosimetry

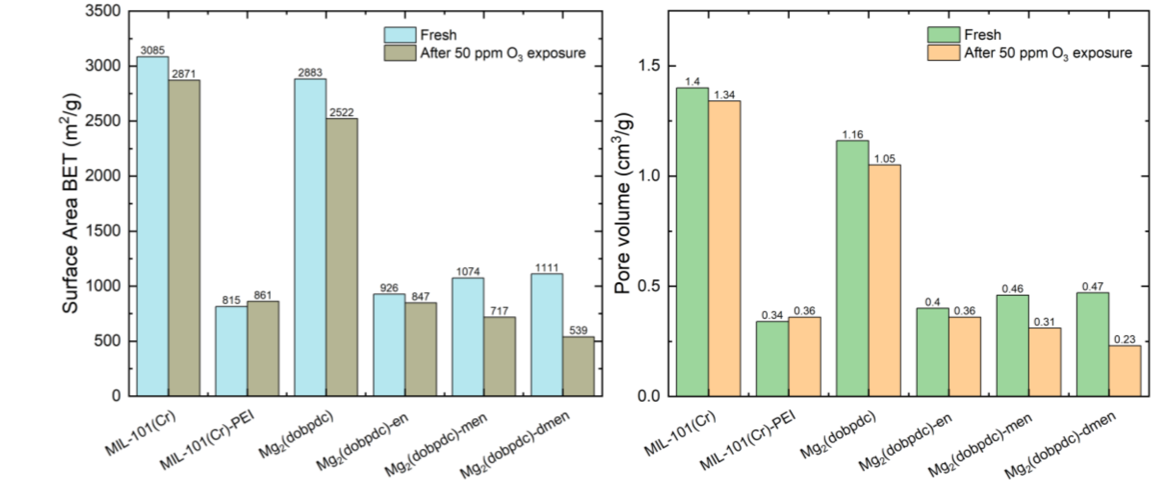

Figure 21. BET surface area (left) and pore volume obtained at P/Po of 0.8 (right) of amine composites before and after 50 ppm O3 exposure.

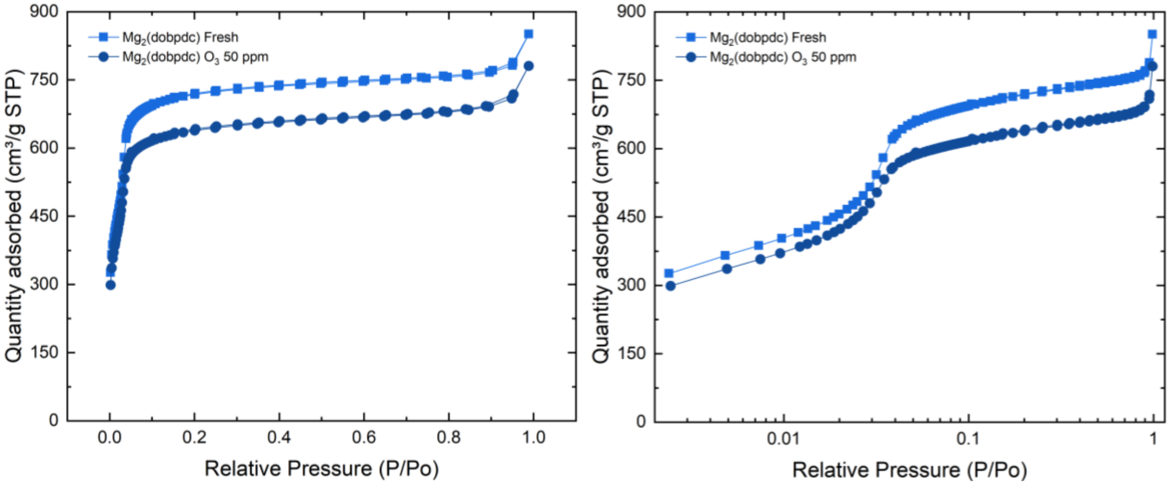

Figure S22. N_2 isotherms at 77 K for fresh Mg_2(dobpdc) (blue) and after exposure to 50 ppm ozone ozone in oxygen-deficient air (dark blue). Logarithmic pressure scale isotherm (right).

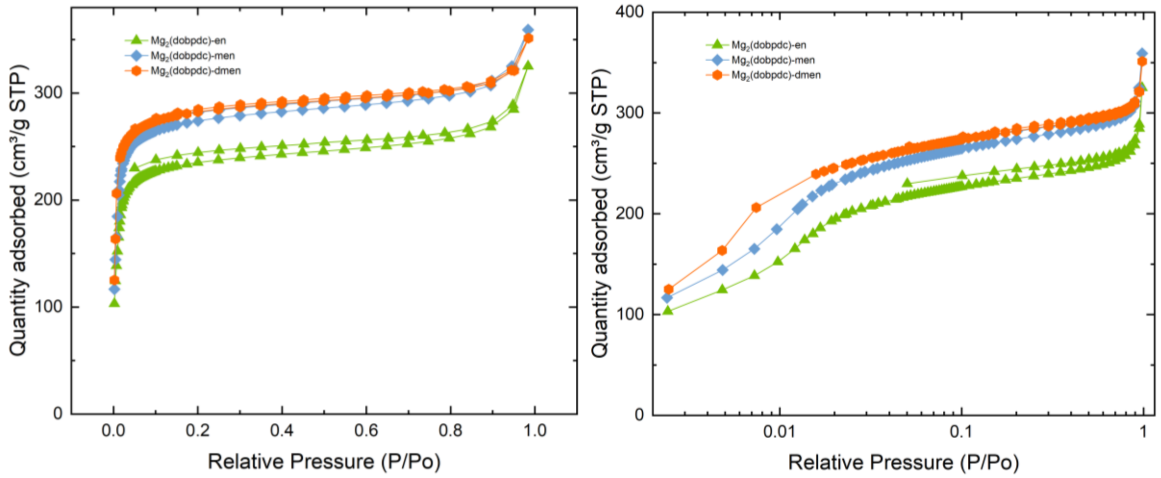

Figure S23. N_2 isotherms at 77 K for fresh Mg_2(dobpdc)-en (green), Mg_2(dobpdc)-men (gray blue), Mg_2(dobpdc)-dmen (orange). Logarithmic pressure scale isotherm (right).

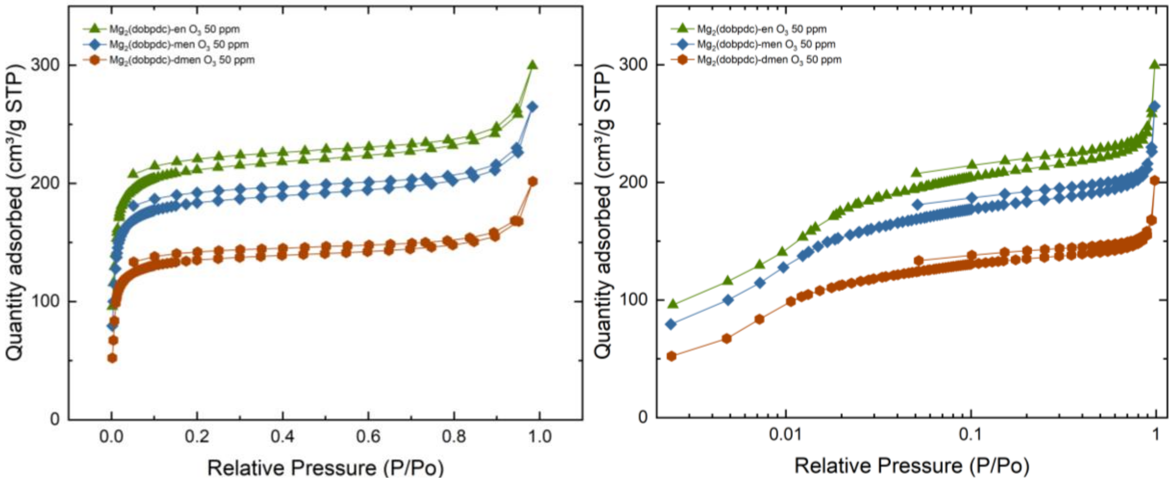

Figure S24. N_2 isotherms at 77 K for fresh Mg_2(dobpdc)-en (dark green), Mg_2(dobpdc)-men (dark gray blue), Mg_2(dobpdc)-dmen (dark orange) after exposure to 50 ppm ozone in oxygen-deficient air. Logarithmic pressure scale isotherm (right).

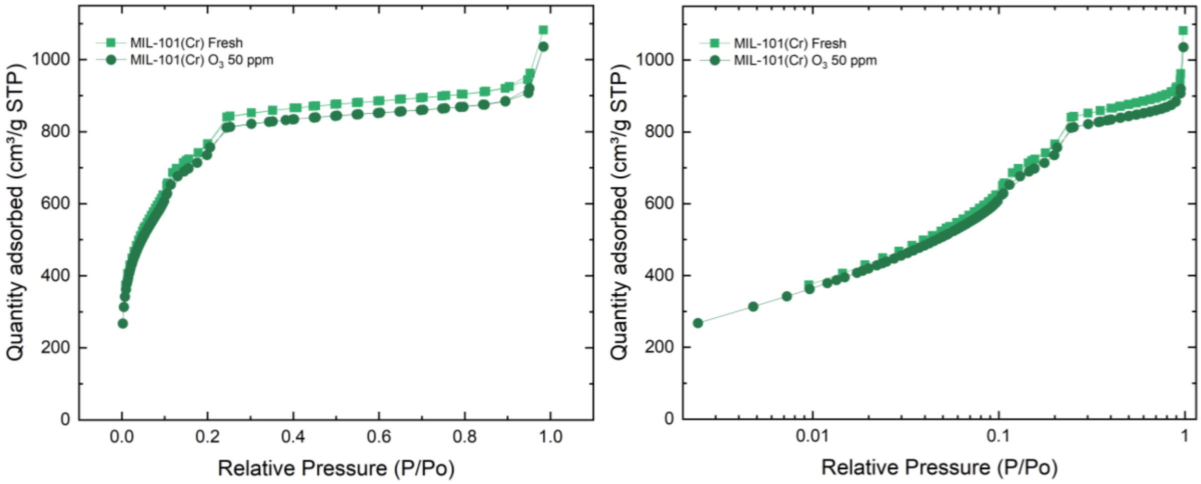

Figure S25. N_2 isotherms at 77 K for fresh MIL-101(Cr) before (bright green) and after exposure to 50 ppm ozone in oxygen-deficient air (dark green). Logarithmic pressure scale isotherm (right).

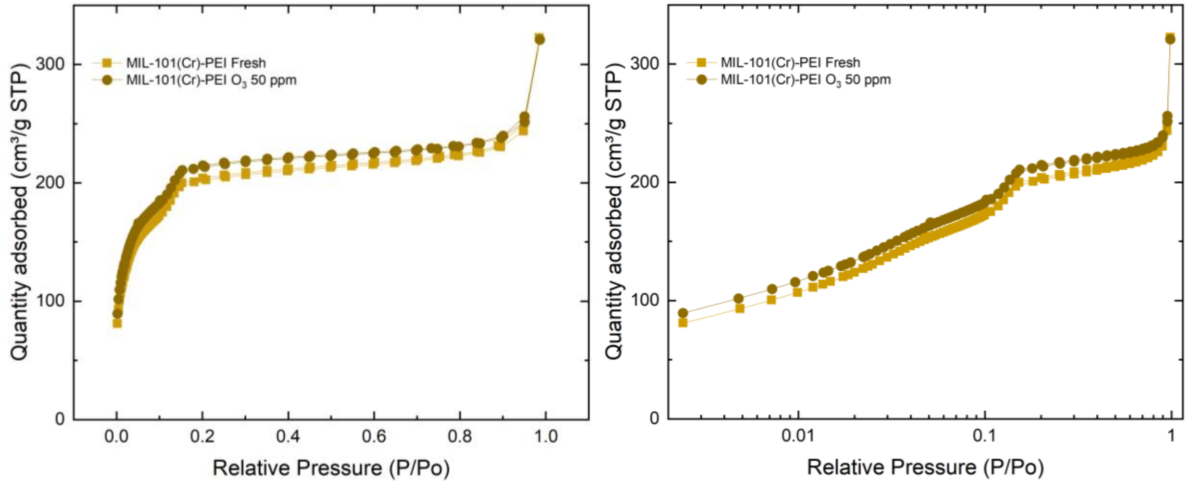

Figure S26. N_2 isotherms at 77 K for fresh MIL-101(Cr)-PEI before (bright yellow) and after exposure to 50 ppm ozone in oxygen-deficient air (dark yellow). Logarithmic pressure scale isotherm (right).

CO_2 adsorption isotherms at 298 K

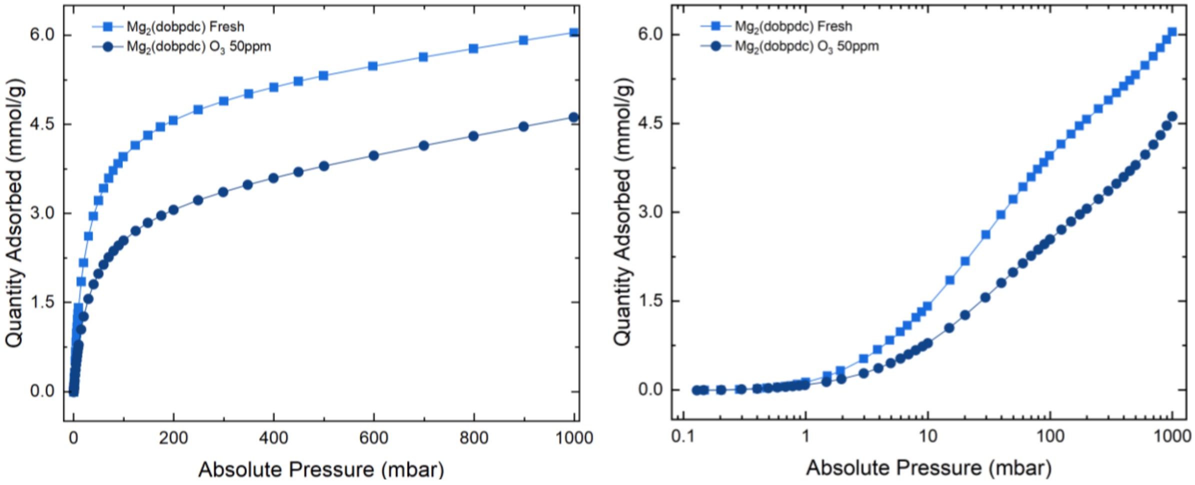

Figure S27. CO_2 isotherm for Mg_2(dobpdc) in its fresh state (blue) and after exposure to 50 ppm of ozone in oxygen-deficient synthetic air (dark blue). Logarithmic pressure scale isotherm (right).

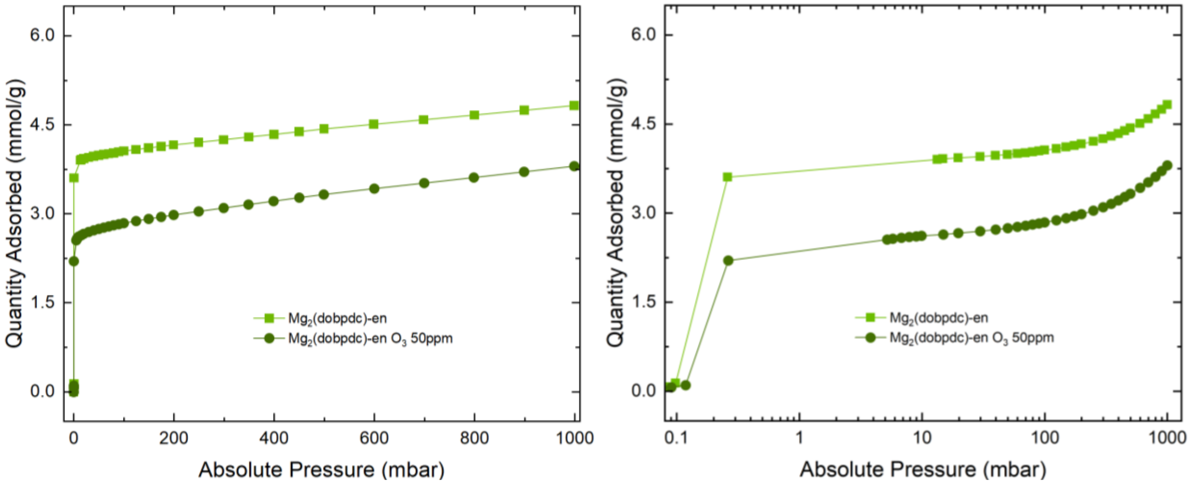

Figure S28. CO_2 isotherm for Mg_2(dobpdc)-en in its fresh state (green) and after exposure to 50 ppm of ozone in oxygen-deficient synthetic air (dark green). Logarithmic pressure scale isotherm (right).

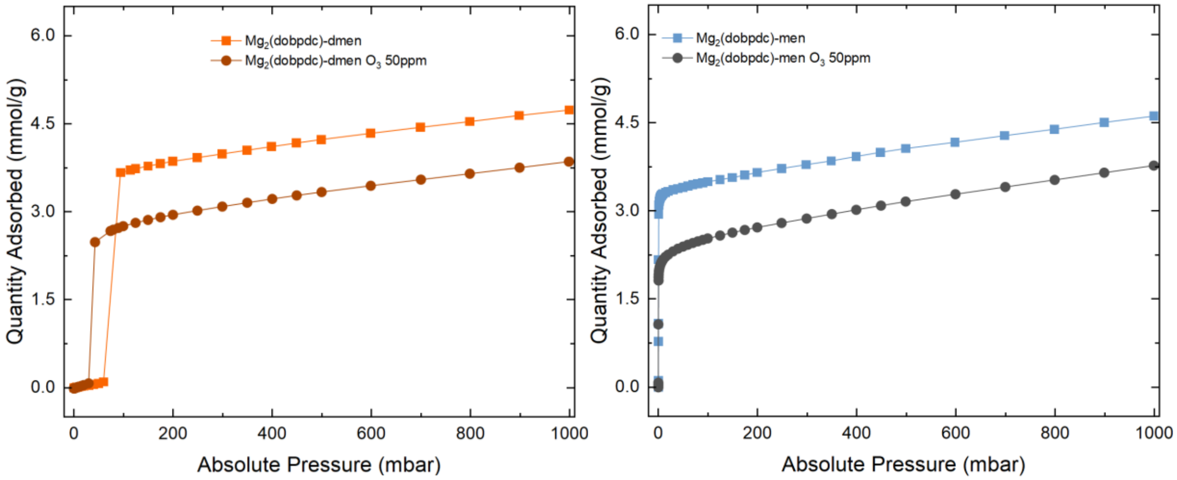

Figure S29. CO_2 isotherms for Mg_2(dobpdc)-dmen (left, orange) and Mg_2(dobpdc)-men in their fresh state (right, blue) and after exposure to 50 ppm of ozone in oxygen-deficient synthetic air (dark orange and dark blue, respectively).

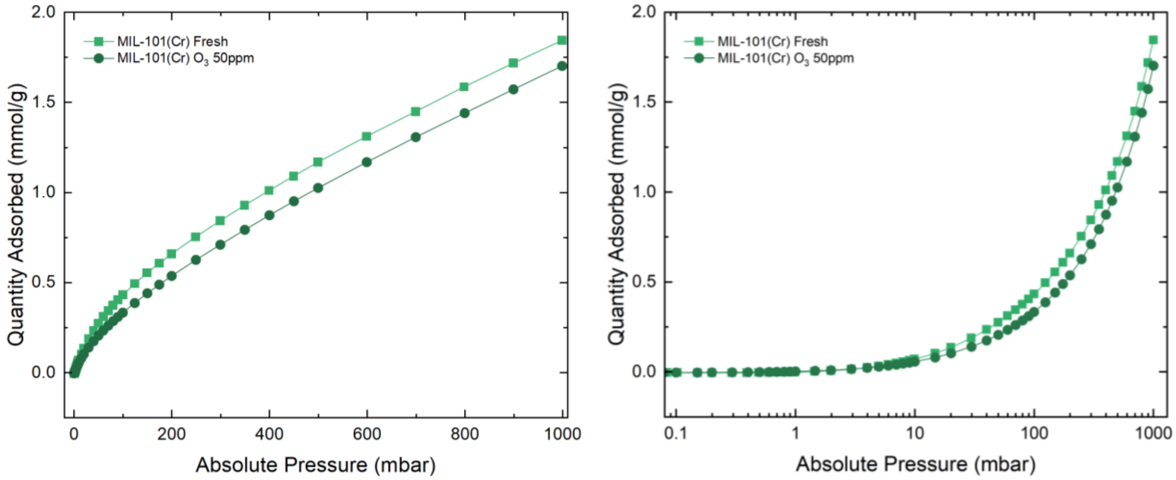

Figure S30. CO_2 isotherm for MIL-101(Cr) in its fresh state (green) and after exposure to 50 ppm of ozone in oxygen-deficient synthetic air (dark green). Logarithmic pressure scale isotherm (right).

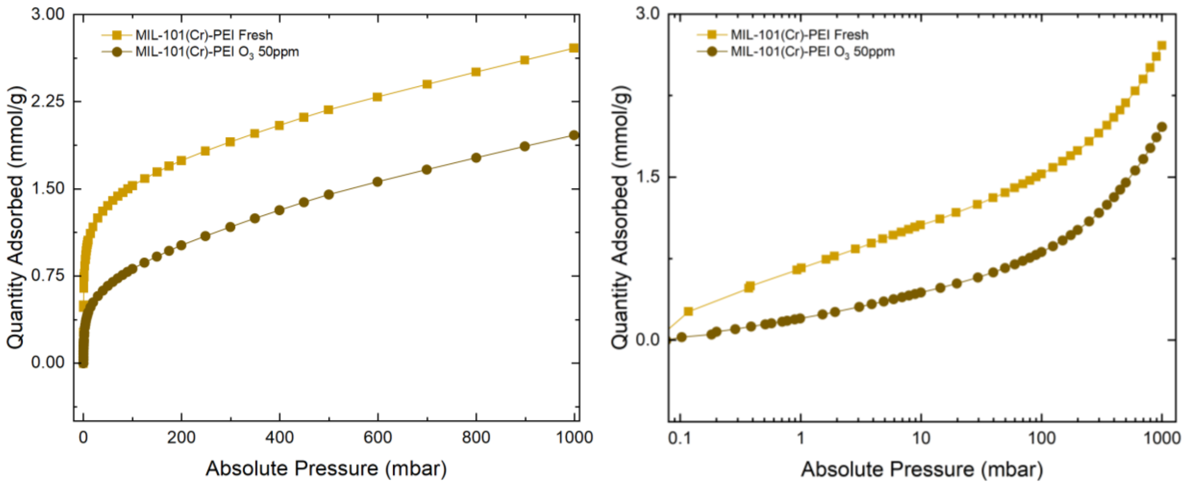

Figure S31. CO_2 isotherm for MIL-101(Cr)-PEI in its fresh state (yellow) and after exposure to 50 ppm of ozone in oxygen-deficient synthetic air (dark yellow). Logarithmic pressure scale isotherm (right).

CO_2 dynamic adsorption measurements with 400 ppm carbon dioxide in N_2 at 298 K

Additional powder diffraction patterns

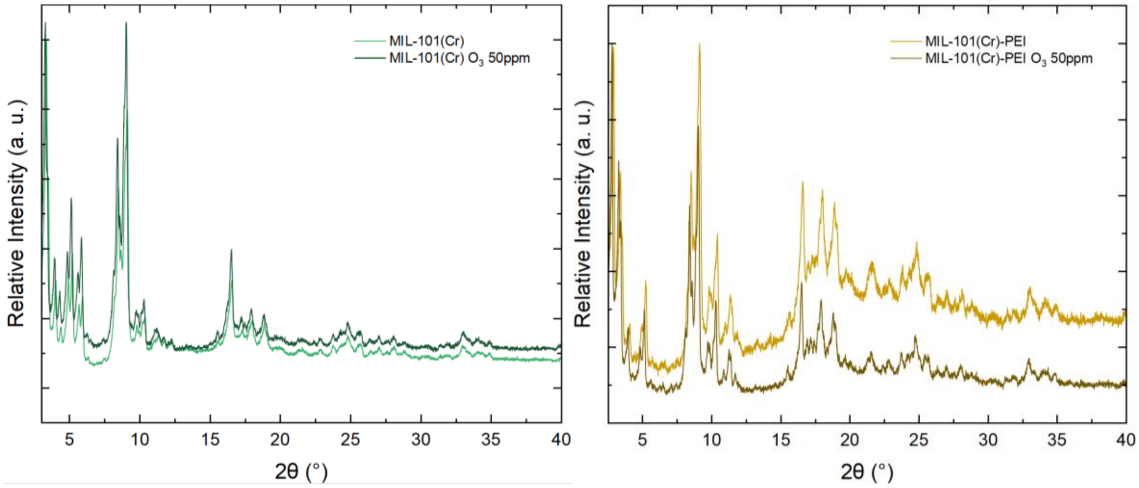

Figure S32. Diffraction patterns of MIL-101(Cr) and MIL-101(Cr)-PEI before and after exposure to oxygen-deficient synthetic air containing 50 ppm of ozone

ATR-FTIR spectra of composites before and after exposure

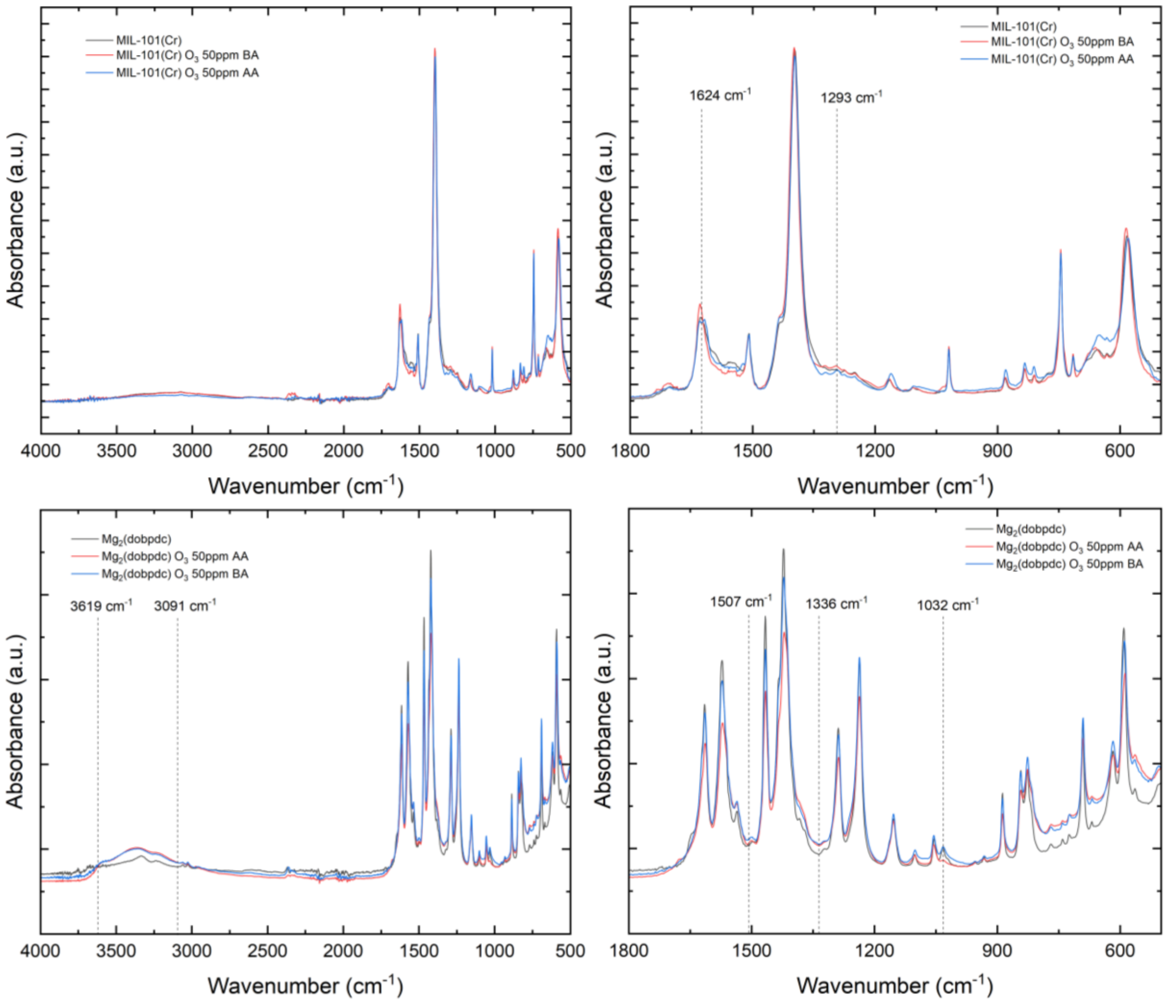

Figure S33. ATR-FTIR spectra of MIL-101(Cr) and Mg_2(dobpdc) MOFs before and after exposure to 50 ppm of ozone. Figures on the right represent a zoom into fingerprint region (500-1800 cm^{-1}).

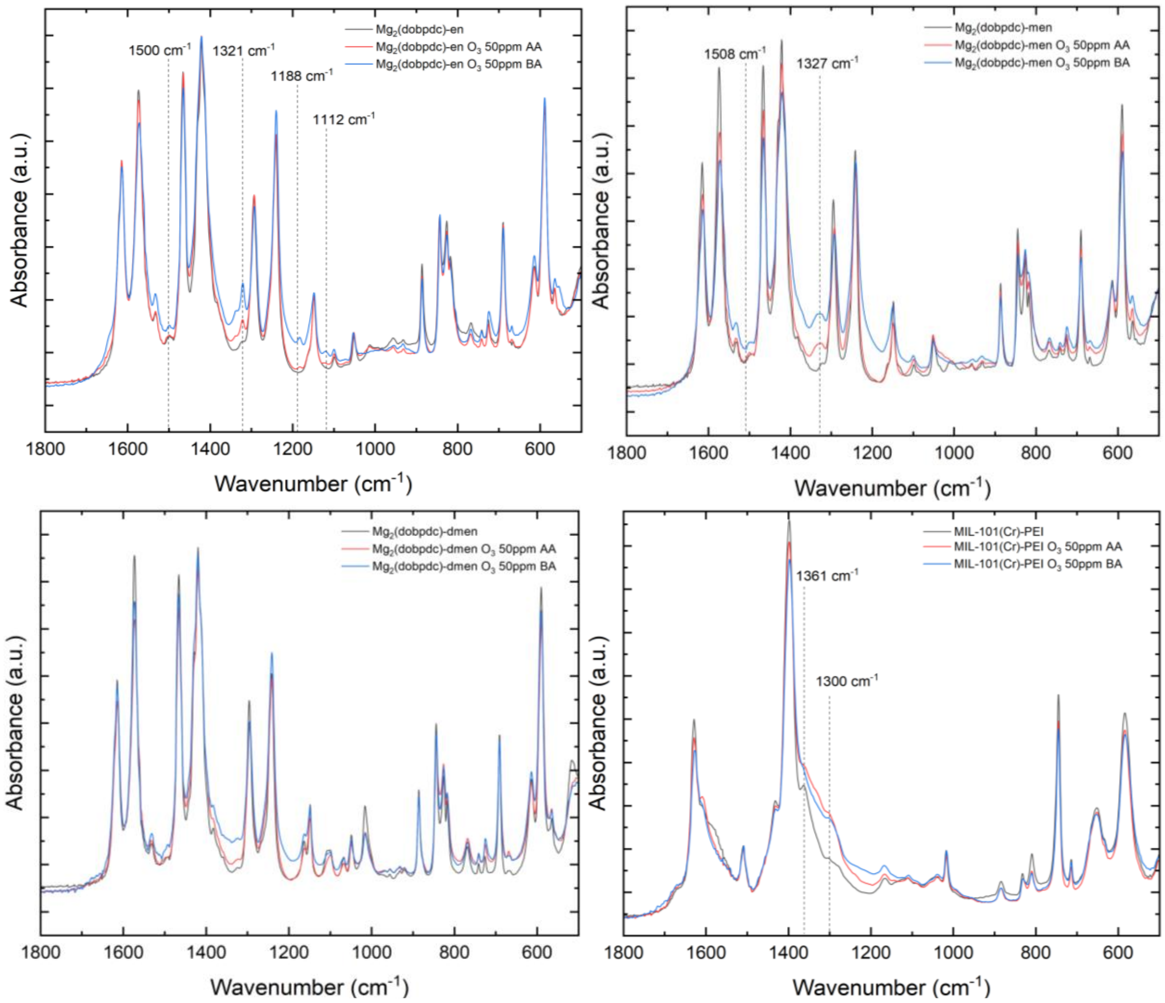

Figure S34. ATR-FTIR spectra at the 500-1800 cm^{-1} fingerprint region of diamine-appended Mg_2(dobpdc) and MIL-101(Cr)-PEI samples before (black), after exposure to oxygen deficient air containing 50 ppm of ozone (blue) and after reactivation (red)

Additionally, two evident changes are observed as small but relatively sharp bands appearing at 1120 cm^{-1} and 1180 cm^{-1} in Mg_2(dobpdc)-en that are reduced in intensity but not lost after reactivation, which are attributed to additional C-O and C=O since the slow reaction between benzene and ozone is possible and expected to be exothermic[6]. In particular, hydroxyl groups present in H_4(dobpdc) may enhance the kinetics of the reaction due to the activation of the aromatic ring, and the electron donation from the metal bound amine may also foster this process[7].

After exposure of MIL-101(Cr)-PEI to O_3 broad bands overlap between 1225-1370 cm^{-1}, which may represent a convolution between vibrational modes of C-NO_2, N-O, N^+-O^- as the presence of nitro moieties and amine oxides as well as N-NO_2 as a contribution from nitrogen oxide impurities. However, it is difficult to deconvolute the potential contributions to state the presence of a certain moiety in this case such as what was observed by Bhattacharyya et. al. after exposure of ZIF-8 to NO_2[8]; thus, changes in this region are attributed to oxidized amines in general.

FTIR spectra of the samples exposed to high concentrations of O_3 confirm the degradation of the samples through the formation of nitro moieties as broad absorbance bands in the regions between

1300-1350 cm^{-1} for MIL-101(Cr)-PEI and Mg_2(dobpdc) diamine-appended and pristine MOF samples and between 1500-1530 cm^{-1} only for Mg_2(dobpdc) diamine-appended and bare MOF samples (Figures S33-S35).

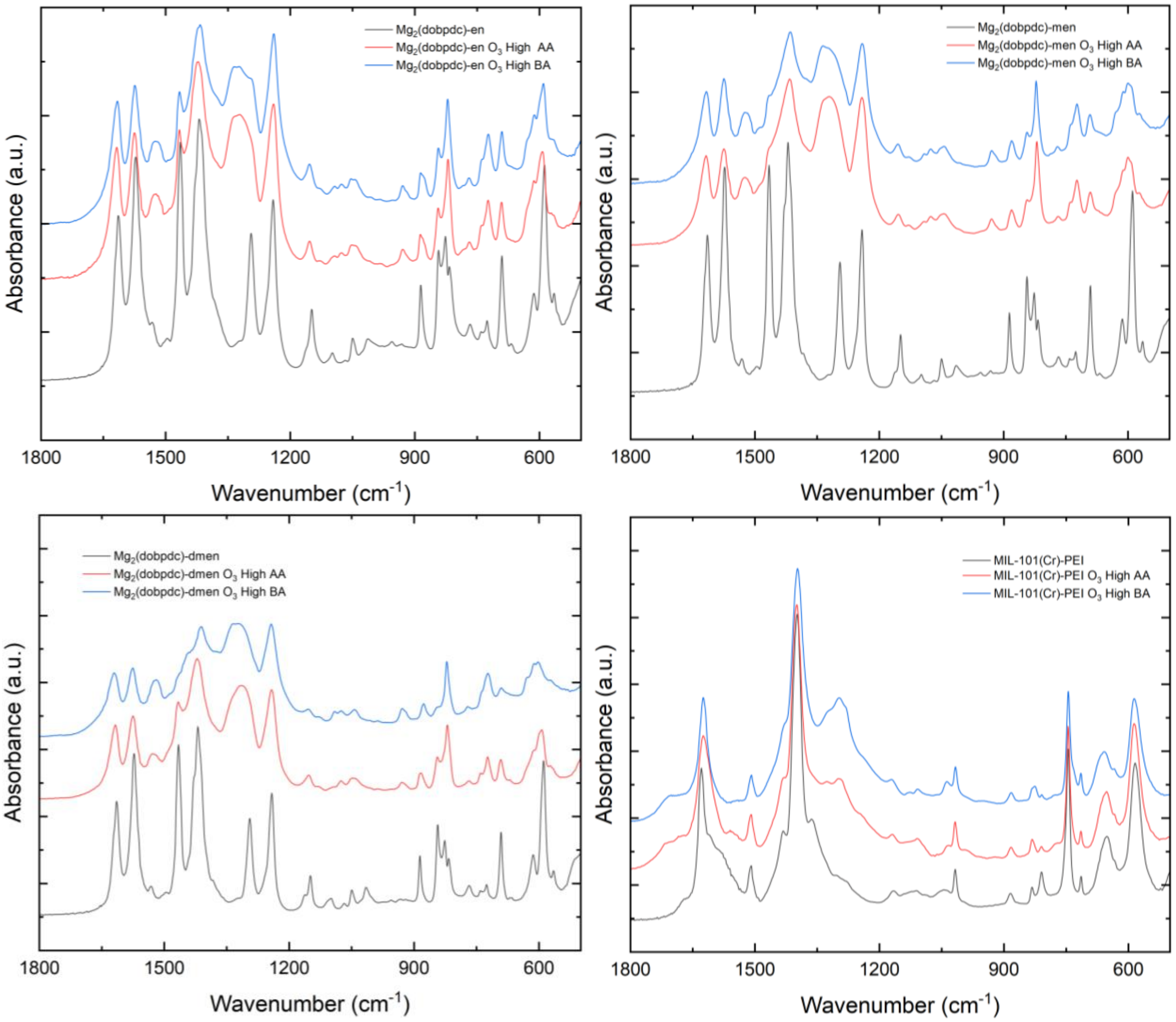

Figure S35. ATR-FTIR spectra at the 500-1800 cm^{-1} fingerprint region of diamine-appended Mg_2(dobpdc) and MIL-101(Cr)-PEI samples before and after exposure to air containing high concentrations of ozone as well as nitrogen oxide byproducts. Main changes are associated with the presence of nitro moieties and potentially nitramines and amine oxides. Offset applied for clarity.

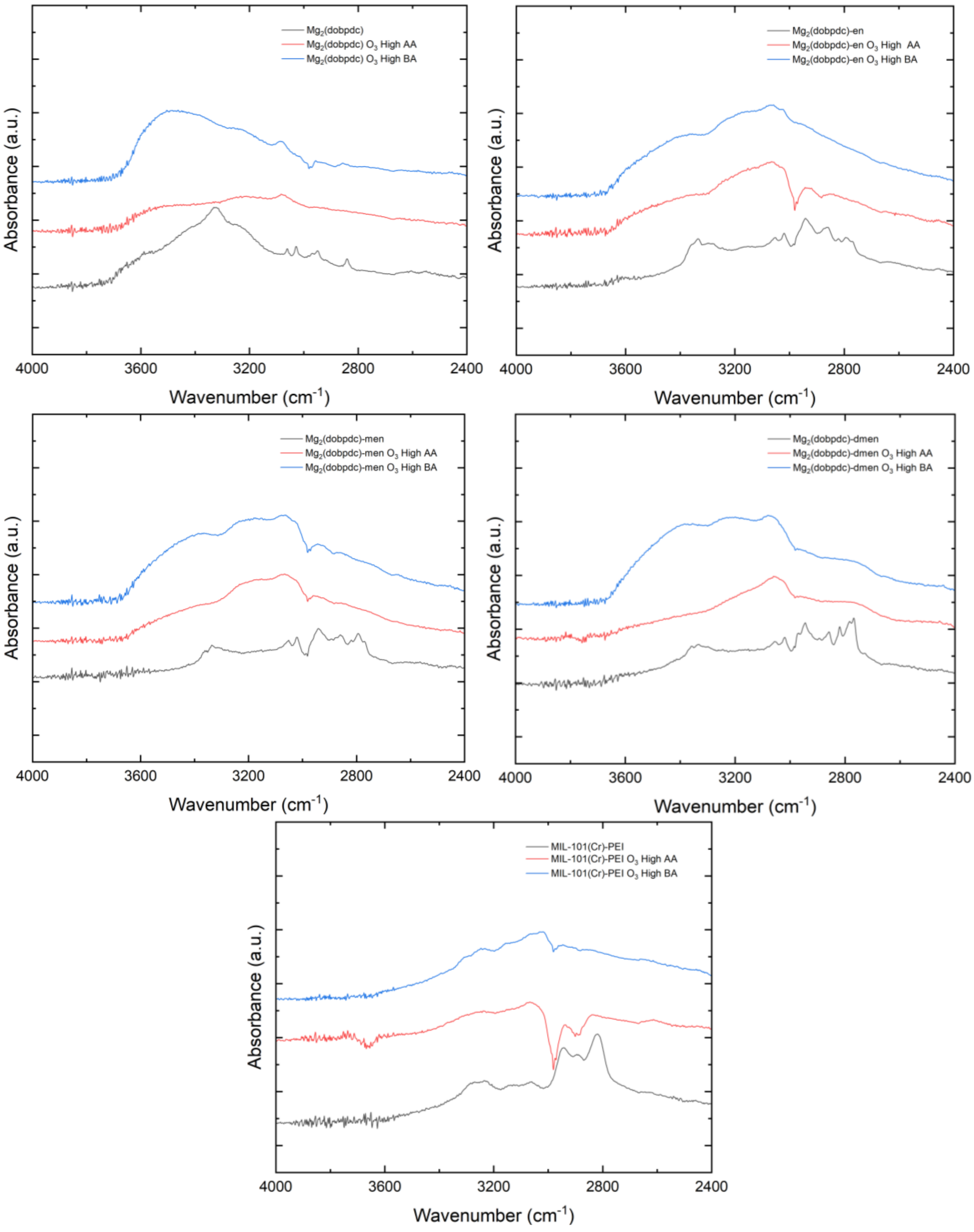

Figure S36. ATR-FTIR spectra at the 2400-4000 cm^{-1} region of diamine-appended Mg_2(dobpdc) and MIL-101(Cr)-PEI samples before and after exposure to air containing high concentrations of ozone as well as nitrogen oxide byproducts. Bands related to C-H stretch of amine molecules are not observed in exposed and reactivated samples. Offset applied for clarity.

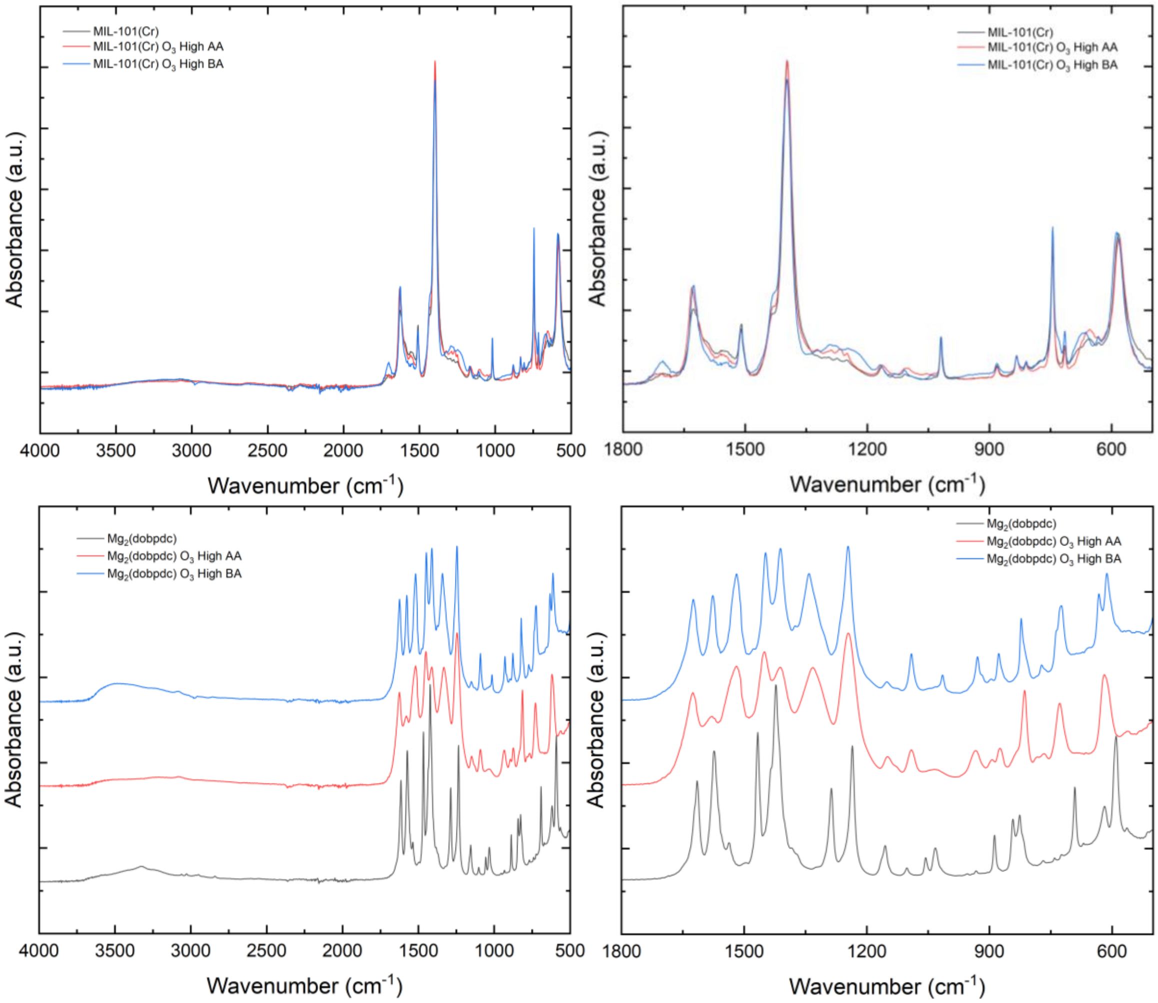

Figure S37. ATR-FTIR spectra of MIL-101(Cr) and Mg_2(dobpdc) before and after exposure to air containing high concentrations of ozone as well as nitrogen oxide byproducts. Figures on the right represent a zoom into fingerprint region (500-1800 cm^{-1}). Offset applied for clarity.

X-ray photoelectron core spectra

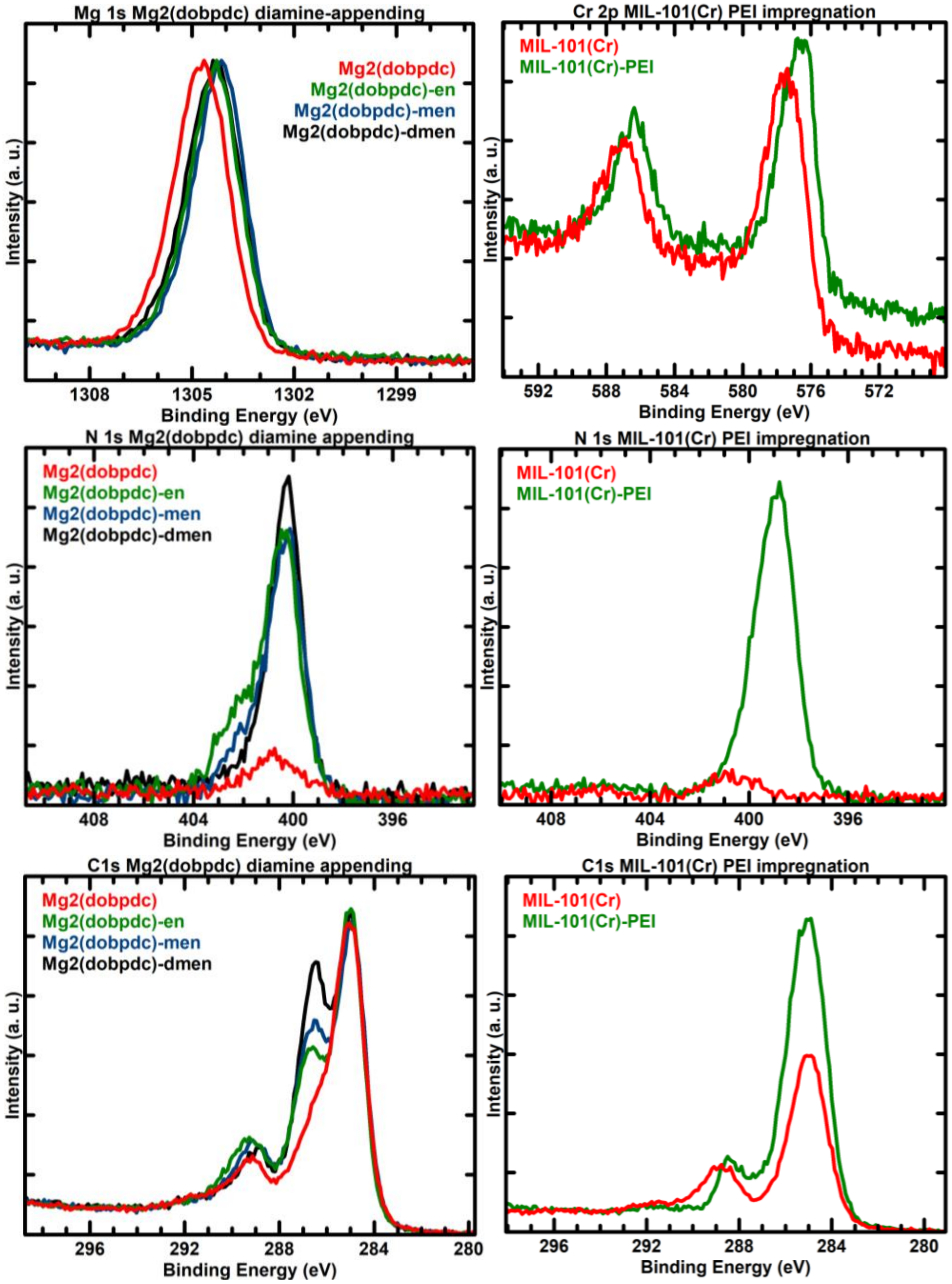

Figure S38. XPS core spectra of amine decorated Mg_2(dobpdc) (left) and MIL-101(Cr) (right) showing a shift towards lower binding energy on the metal XPS spectra (top) for both MOFs after post-synthetic modification with amines. N 1s (center) and C 1s (bottom) XPS scans of amine grafted Mg2(dobpdc) and impregnated MIL-101(Cr) show the presence of amines in the composites.

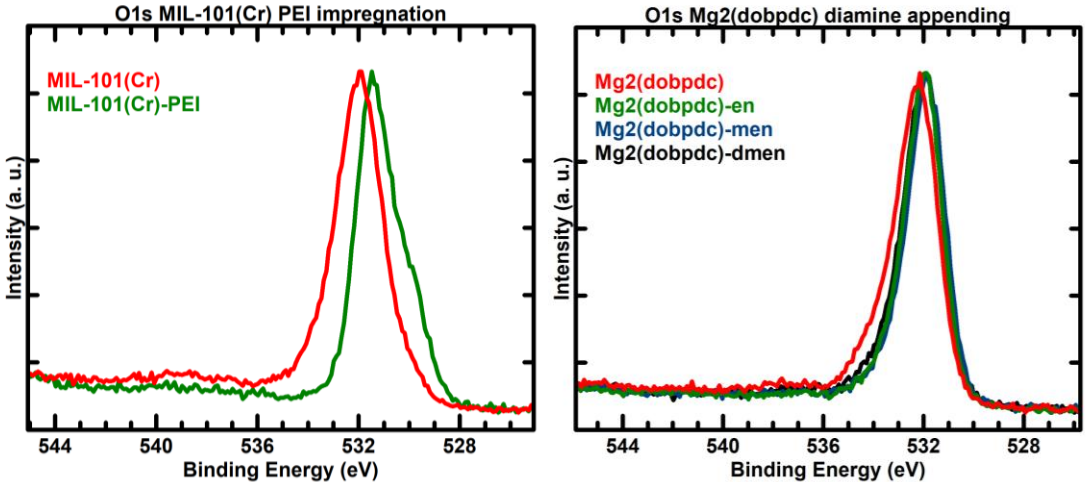

Figure S39. XPS core spectra comparing fresh samples of MIL-101(Cr) and MIL-101(Cr)-PEI (left), and of Mg_2(dobpdc) samples before and after diamine grafting (right). Shift in BE of O 1s peak indicates electron donation from amines to metal sites since it is representative of the oxygen atoms from carboxylate groups in the ligands of the MOFs.

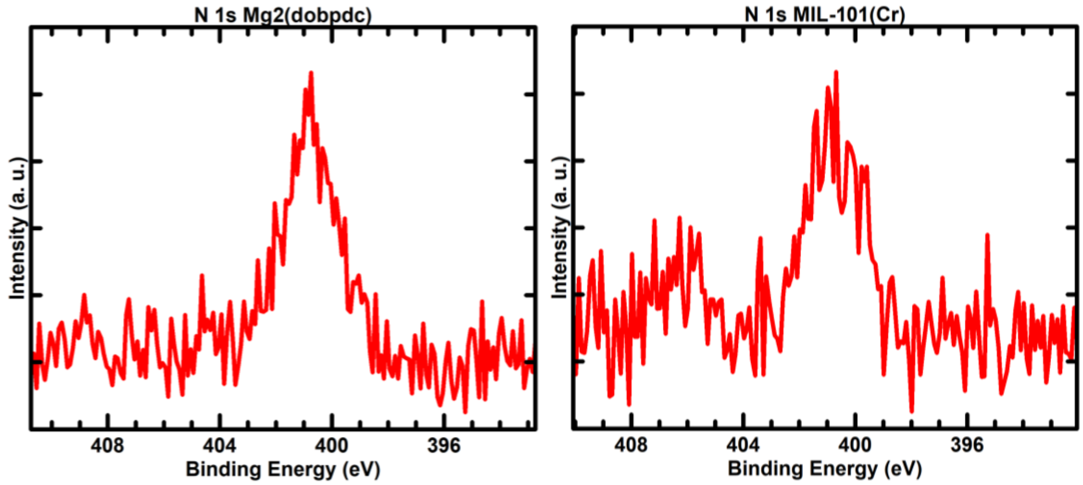

Figure S40. N1s core spectra fresh samples of Mg_2(dobpdc) and MIL-101(Cr).

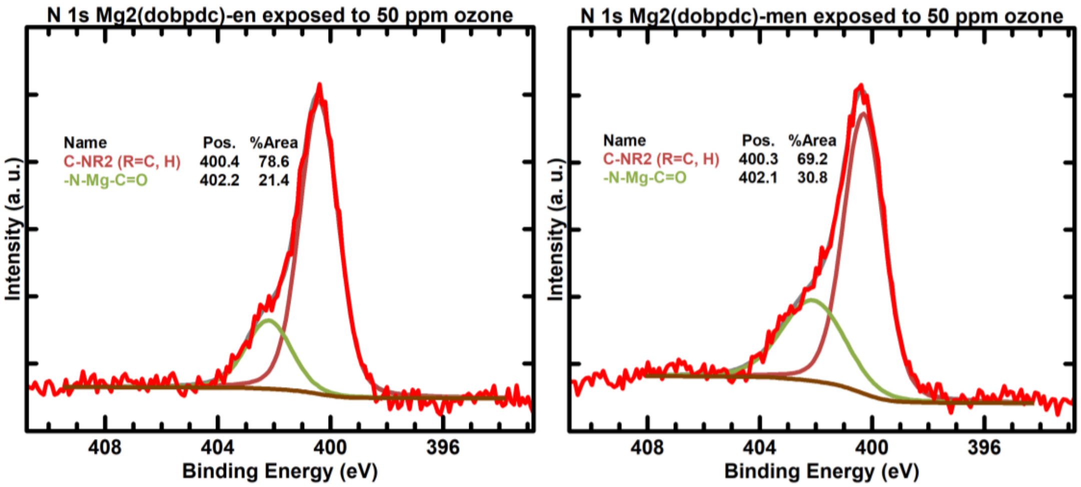

Figure S41. N1s core spectra of Mg_2(dobpdc)-en and Mg_2(dobpdc)-men after exposure to 50 ppm of O_3 in oxygen-deficient air.

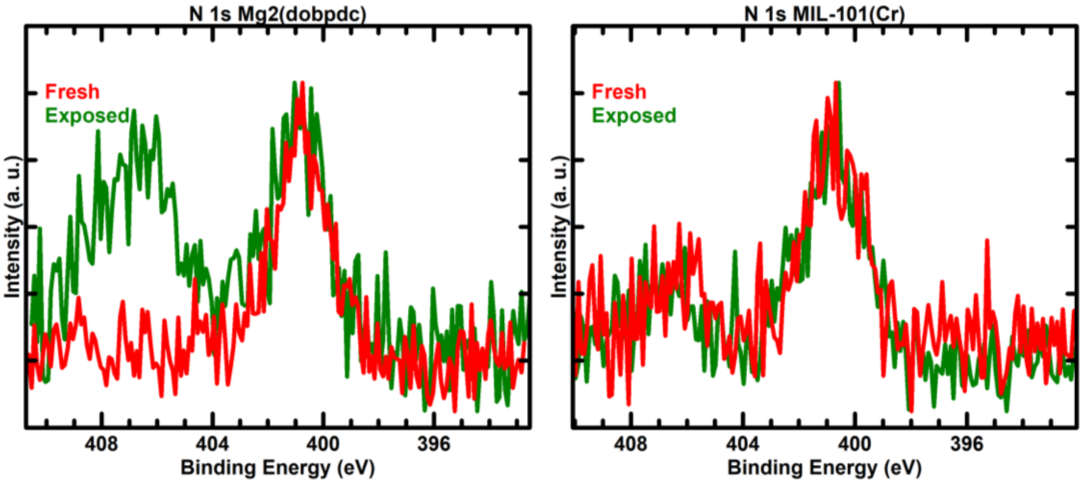

Figure S42. N1s core spectra of Mg_2(dobpdc) (left) and MIL-101(Cr) (right) after exposure to 50 ppm of O_3 in oxygen deficient air. Changes in Mg_2(dobpdc) are attributed to adsorbed NOx or oxidized residual solvent.

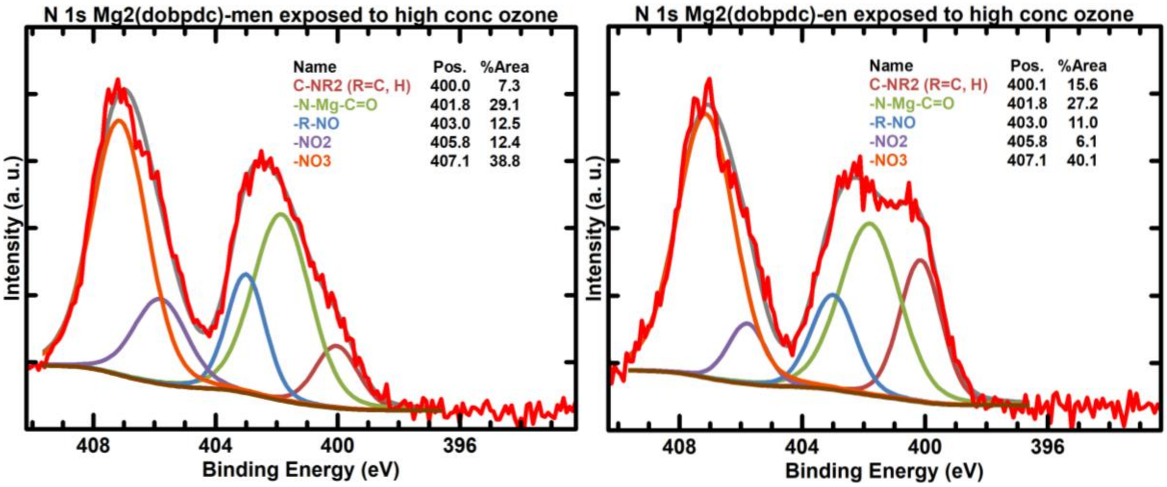

Figure S43. N 1s XPS spectra of Mg_2(dobpdc)-en (left) and Mg_2(dobpdc)-men (right) after exposure to high concentrations of O_3. Mg_2(dobpdc)-men shows a higher difference between the peak assigned to amine groups and the rest of the oxidation products compared to Mg_2(dobpdc)-en.

Table S1. Elemental compositions from fitted regions on high resolution XPS core scans for samples exposed to 50 ppm ozone show varying degrees of change in nitrogen and oxygen atomic percentages.

	Mg_2(dobpdc)		Mg_2(dobpdc)-en		Mg_2(dobpdc)-men		Mg_2(dobpdc)-dmen		MIL-101(Cr)		MIL-101(Cr)-PEI	
**Element**	**Fresh**	**Exposed**	**Fresh**	**Exposed**	**Fresh**	**Exposed**	**Fresh**	**Exposed**	**Fresh**	**Exposed**	**Fresh**	**Exposed**
**C**	58.67	56.94	55.33	56.38	56.56	55.90	59.02	56.67	60.20	60.43	60.14	59.66
**M**	13.21	11.33	10.66	10.88	10.46	10.77	11.29	10.32	4.73	4.50	3.07	3.13
**N**	1.94	3.98	10.08	8.97	10.60	10.08	8.82	9.49	2.23	2.47	16.58	15.71
**O**	26.17	27.74	23.92	23.77	22.38	23.24	20.86	23.52	32.84	32.59	20.21	21.50
**N/M**	0.15	0.35	0.95	0.82	1.01	0.94	0.78	0.92	0.47	0.55	5.40	5.02

**O/M**	1.98	2.45	2.24	2.18	2.14	2.16	1.85	2.28	6.94	7.24	6.58	6.87
**% change N/M**		+139%		-13%		-8%		+18%		+16%		-7%
**% change O/M**		+24%		-3%		+1%		+23%		+4%		+4%

Table S2. Elemental composition in terms of atomic percentage for each sample of the set of experiments performed at full capacity of the ozone generator. Oxygen to metal (O/M), and nitrogen to metal (N/M) ratios and percent changes are presented. Nitrogen to oxygen ratio respective to the change is presented as a calculation for the increase in oxygen atoms per change in nitrogen atoms.

	**Mg_2(dobpdc)**		**Mg_2(dobpdc)-en**		**Mg_2(dobpdc)-men**		**Mg_2(dobpdc)-dmen**		**MIL-101(Cr)**		**MIL-101(Cr)-PEI**	
**Element**	**Fresh**	**Exposed**	**Fresh**	**Exposed**	**Fresh**	**Exposed**	**Fresh**	**Exposed**	**Fresh**	**Exposed**	**Fresh**	**Exposed**
**C**	57.61	48.32	56.42	49.24	57.56	46.55	57.67	48.38	62.30	59.95	61.75	56.82
**M**	13.87	10.20	10.67	8.76	10.69	7.95	11.47	8.06	4.71	4.19	2.97	2.99
**N**	2.40	8.86	11.07	11.76	9.50	12.71	9.46	11.98	2.06	4.56	15.82	13.37
**O**	26.13	32.62	21.84	30.24	22.24	32.79	21.41	31.58	30.93	31.3	19.46	26.82
**N/M**	0.17	0.87	1.04	1.34	0.89	1.60	0.83	1.49	0.44	1.09	5.33	4.47
**O/M**	1.88	3.20	2.05	3.45	2.08	4.12	1.87	3.92	6.56	7.47	6.56	8.96
**% change N/M**	+402%		+29%		+80%		+80%		+149%		-16%	
**% change O/M**	+70%		+68%		+98%		+110%		+14%		+37%	
**ΔO/ΔN**	1.89		4.61		2.88		3.10		1.72		2.78	

Table S3. Assignment of binding energies for N 1s XPS spectra

		Binding energy	
Peak	Sample	This work	Reference
C-C, C-H	Mg_2(dobpdc) fresh and exposed to high ozone	284.9	285[9] (Graphitic/Graphene/Carbon nanotube)
C-O, C-N		286.2-286.3	286.2 eV[10] (Graphite exposed to Ar/N_2+NO plasmas)
O-C=O		288.9-289.1	288.7 eV[11] (ref C-C: 284.7 eV carbon coated Si particles)
Pi-Pi* satellite (aromatic rings)		290.5-291	290.91 eV[9] (Graphitic/Graphene/Carbon nanotube)
C-NR2	MIL-101(Cr)-PEI<br>Mg_2(dobpdc)-en<br>Mg_2(dobpdc)-men<br>Mg_2(dobpdc)-dmen	398.9<br>400.2<br>400.2<br>400.2	399.12 eV[12] (NH_2 [$amineH^+Br^-$]-Ac-NH-Cr-BDC)

-N-M (metal bound amine)	MIL-101(Cr)-PEI<br>Mg_2(dobpdc)-en<br>Mg_2(dobpdc)-men<br>Mg_2(dobpdc)-dmen	400.2<br>402.1<br>401.5<br>401.3	N-C=O<br>400.12 eV[12] ([amineH$^+$Br$^-$]-Ac-NH-Cr-BDC)<br>400-401 eV[13] (MOF808-EDTA-TREN)<br>C=(NH)$^+$-C<br>401.2 eV[14] (PCAT)
N-C-NO	Mg_2(dobpdc)-dmen 50 ppm ozone	403.2 eV	401.9-404.6 eV (adsorbed NO in Fe_2O_3)
NO_2	Mg2(dobpdc) and diamine appended high ozone<br>MIL-101(Cr)-PEI	405.8 – 405.8 eV<br>406.7 eV	406-407 eV[15] (NO_2 in 4-nitrobenzaldimine)
NO_3	Mg_2(dobpdc)-dmen 50 ppm ozone<br>Mg_2(dobpdc) and diamine appended high ozone	407.0 eV<br>407.1 eV	407.3 eV[16] ($MgNO_3$)

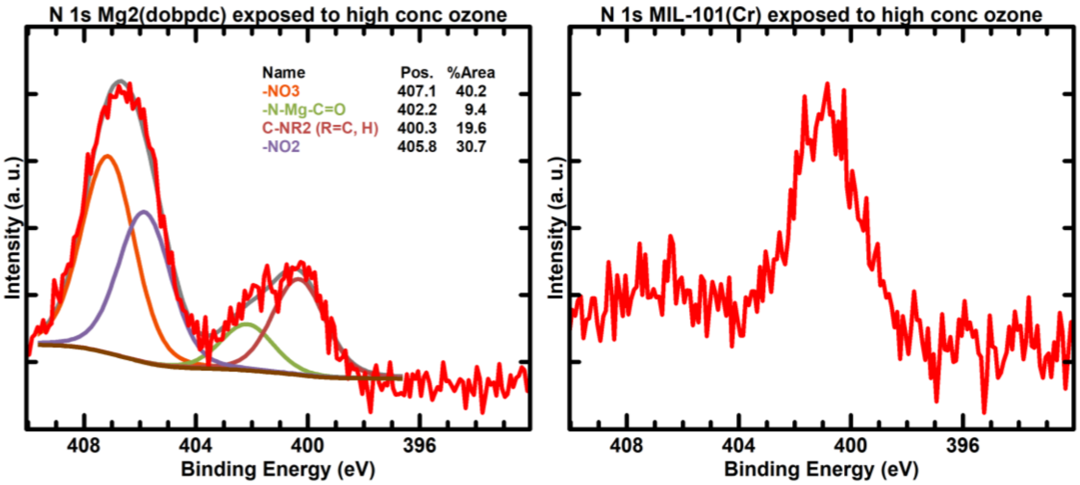

Figure S44. N1s core spectra of Mg_2(dobpdc) and MIL-101(Cr) exposed to air containing high concentration of O_3. The peak model from previous fits was modified by adding nitro and nitrate moieties as components.

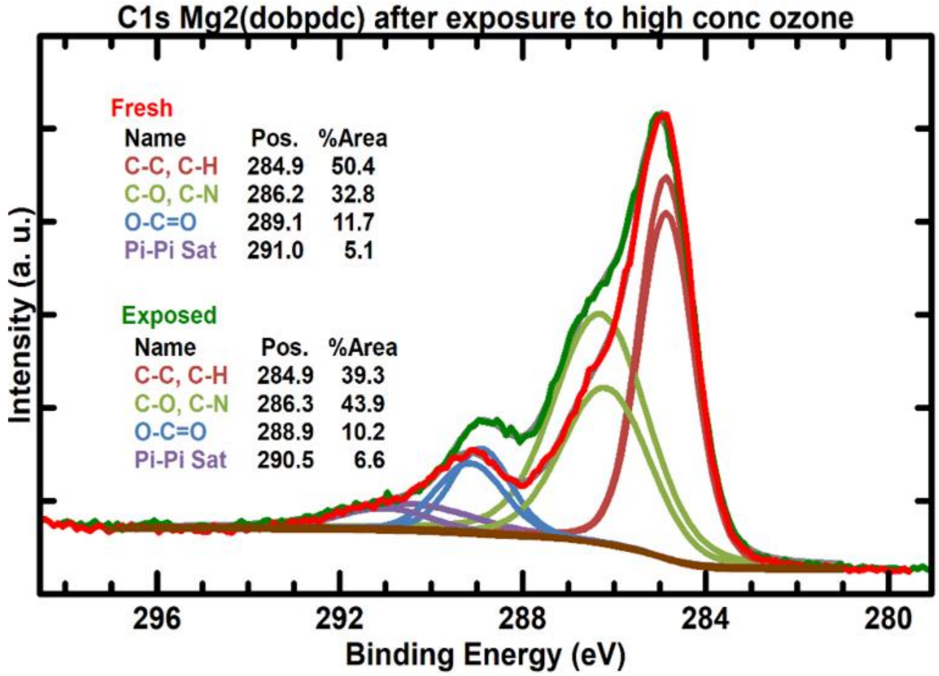

Figure S45. C1s core spectra of Mg_2(dobpdc) exposed to air containing high concentration of O_3 showing an increase in C-O, C-N, which suggests interaction between ozone and the ligand and NOx adsorption.

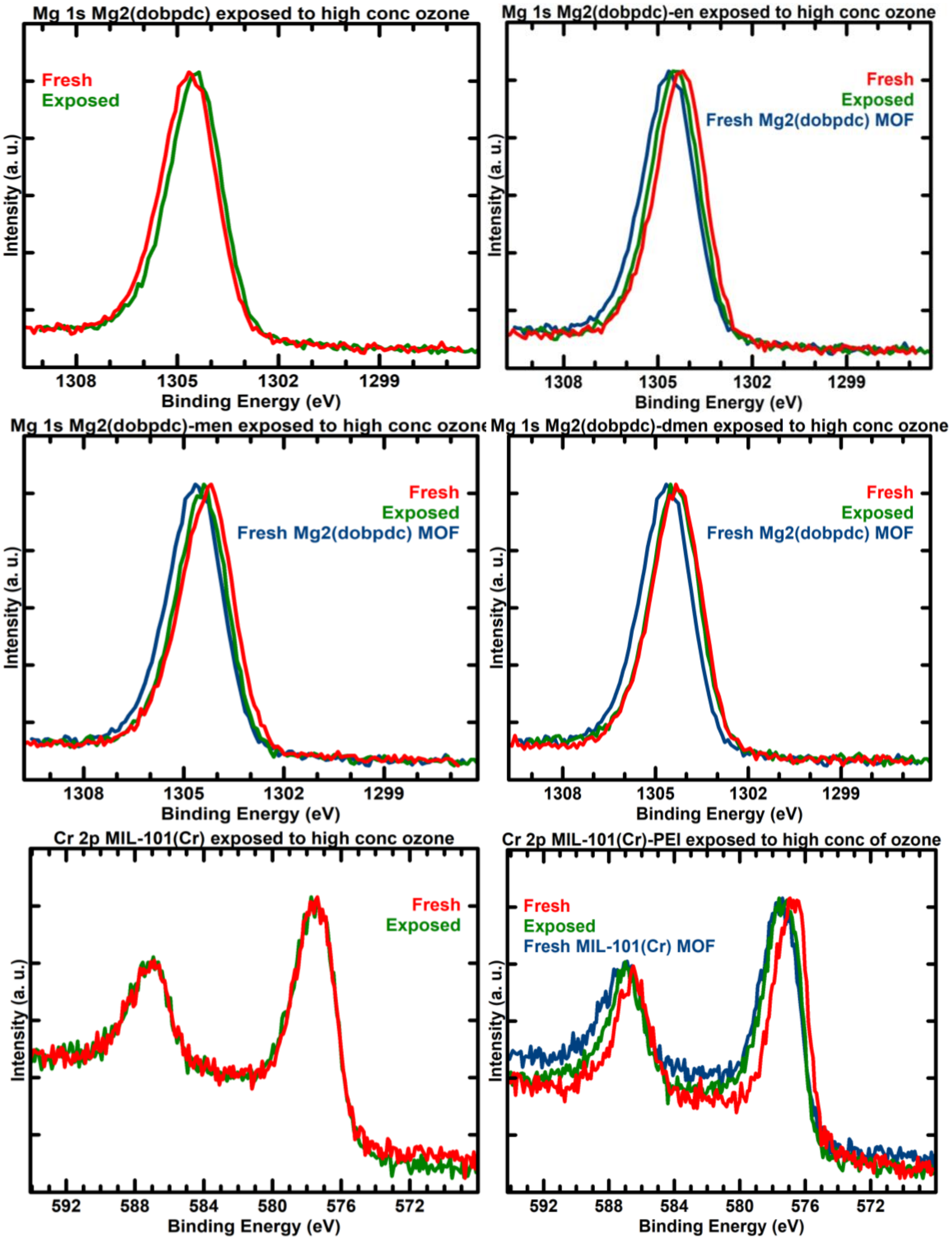

Figure S46. Mg1s and Cr2p core spectra of bare Mg_2(dobpdc), MIL-101(Cr) and diamine-appended Mg_2(dobpdc) samples and MIL-101(Cr)-PEI exposed to air containing high concentrations of O_3. Most amine appended samples show a positive shift after exposure to O_3, which is more significant for MIL-101(Cr)-PEI.

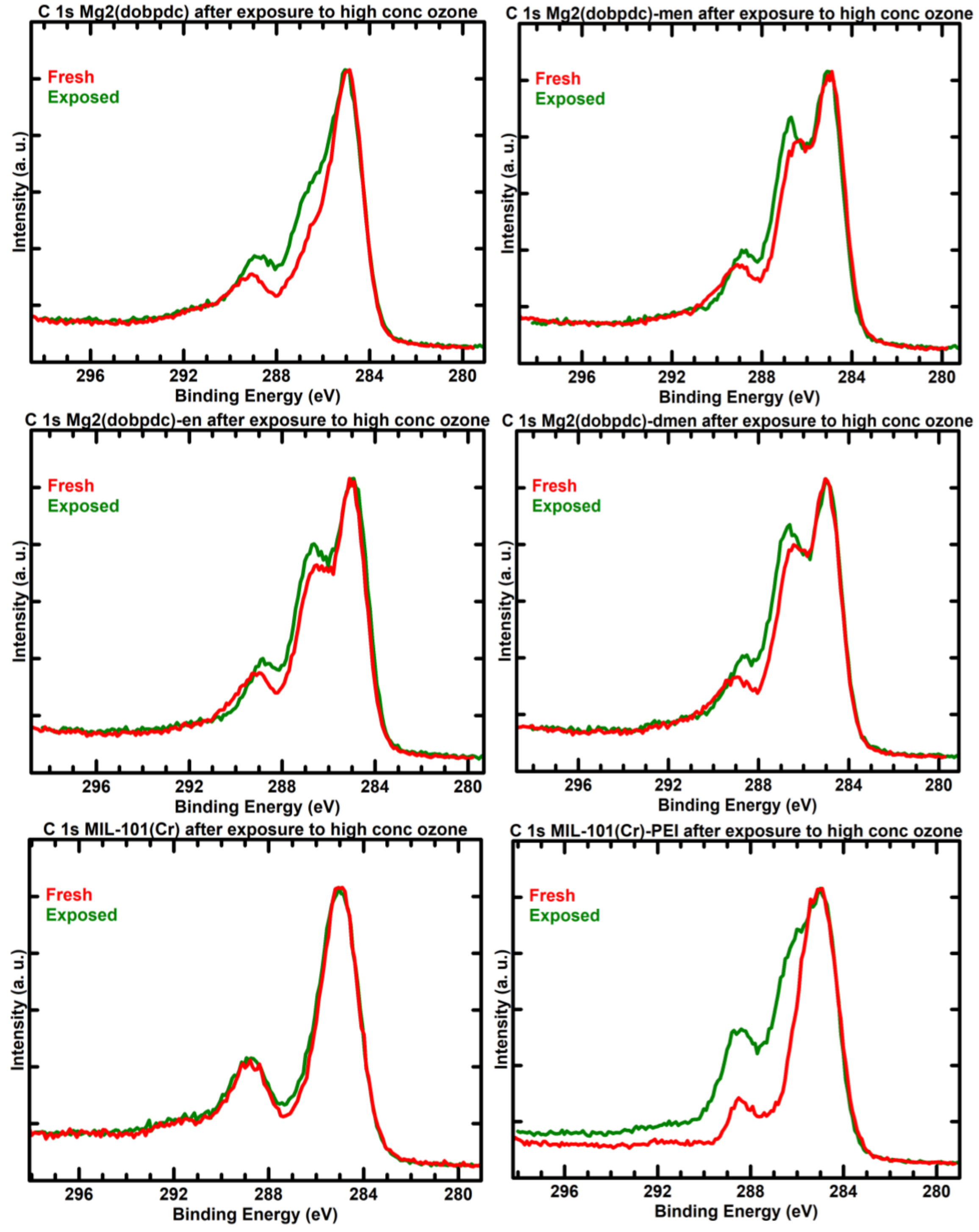

Figure S47. C1s core spectra of bare Mg_2(dobpdc), MIL-101(Cr) and diamine-appended Mg_2(dobpdc) samples and MIL-101(Cr)-PEI exposed to air containing high concentrations of O_3.

Elemental analysis flask combustion CHN

Table S4. Flask combustion CHN analysis of fresh samples and materials exposed to 50 ppm ozone

	Mg_2(dobpdc)		Mg_2(dobpdc)-en		Mg_2(dobpdc)-men		Mg_2(dobpdc)-dmen		MIL-101(Cr)		MIL-101(Cr)-PEI	
**Element**	**Fresh**	**Exposed**	**Fresh**	**Exposed**	**Fresh**	**Exposed**	**Fresh**	**Exposed**	**Fresh**	**Exposed**	**Fresh**	**Exposed**
**C**	44.63	42.40	47.97	45.34	48.35	48.28	51.49	50.75	43.51	41.12	48.00	47.65
**H**	3.95	4.20	5.12	4.77	5.44	4.97	6.09	5.73	2.66	2.90	5.60	5.41
**N**	0.92	1.69	10.70	9.35	10.04	10.89	10.30	9.85	1.78	1.41	13.08	13.26

Table S5. Flask combustion CHN analysis of fresh samples and materials exposed to high concentration of ozone

	Mg_2(dobpdc)		Mg_2(dobpdc)-en		Mg_2(dobpdc)-men		Mg_2(dobpdc)-dmen		MIL-101(Cr)		MIL-101(Cr)-PEI	
**Element**	**Fresh**	**Exposed**	**Fresh**	**Exposed**	**Fresh**	**Exposed**	**Fresh**	**Exposed**	**Fresh**	**Exposed**	**Fresh**	**Exposed**
**C**	43.09	34.33	46.88	36.26	48.36	35.79	49.79	36.26	43.51	41.88	48.00	41.92
**H**	4.33	2.44	5.29	4.23	5.56	3.65	6.44	3.87	2.66	2.98	5.60	4.17
**N**	0.20	7.33	10.17	11.38	9.38	12.27	9.87	11.52	1.78	2.54	13.08	13.48

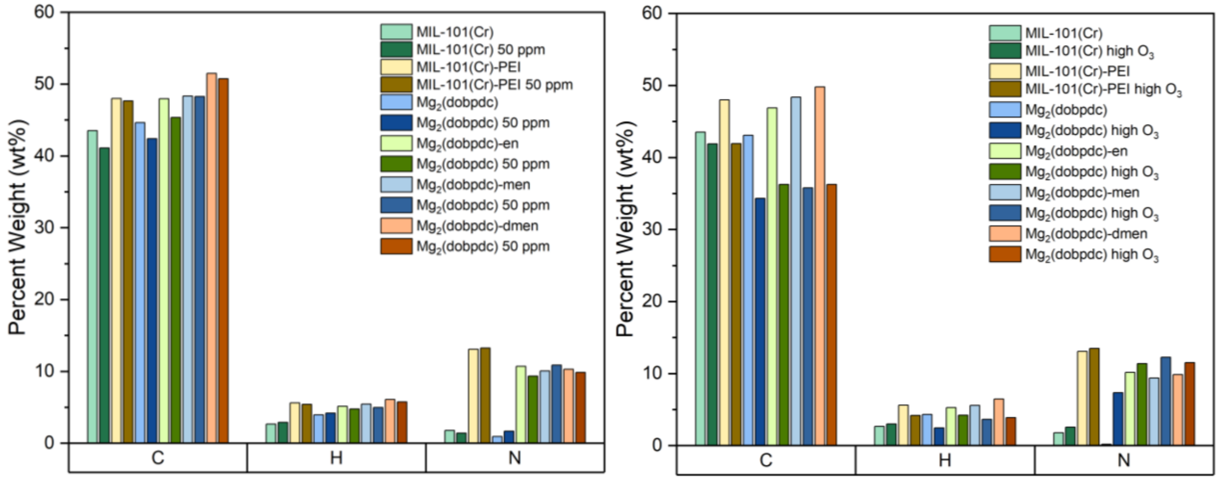

Figure S48. Flask combustion CHN elemental analysis of composites exposed to 50 ppm (left) and high O_3 concentrations (right). Exact values are summarized in Table S3 and Table S4 in supporting information.

C 1s 285 eV referencing

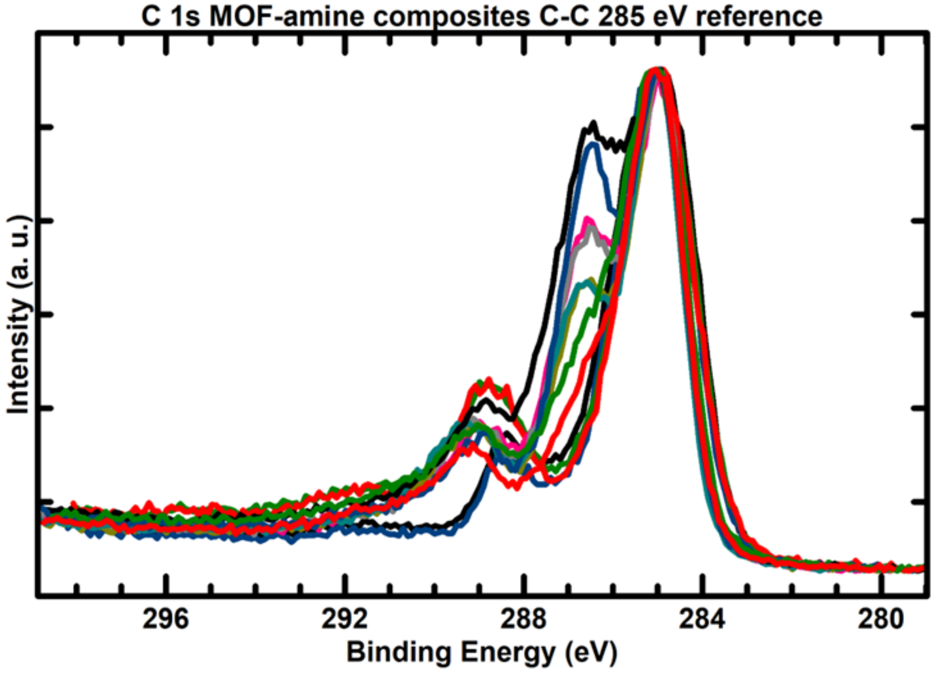

Figure S49. C 1s 285 eV referencing fresh MOF-amine composites and MOF-amine composites exposed to high concentrations of ozone

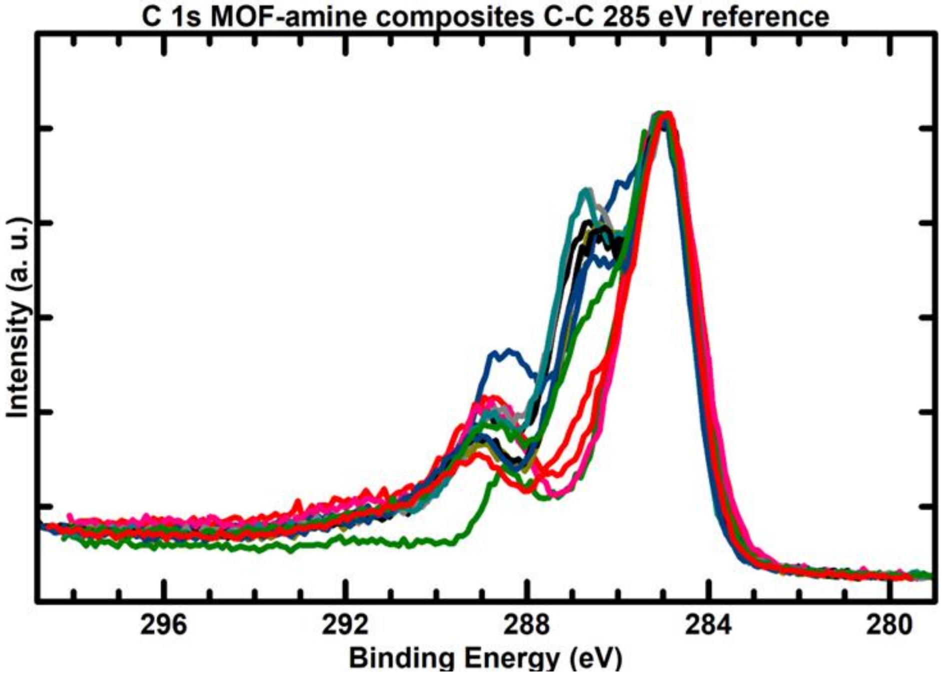

Figure S50. C 1s 285 eV referencing fresh MOF-amine composites and MOF-amine composites exposed to high concentrations of ozone

Thermogravimetric analysis

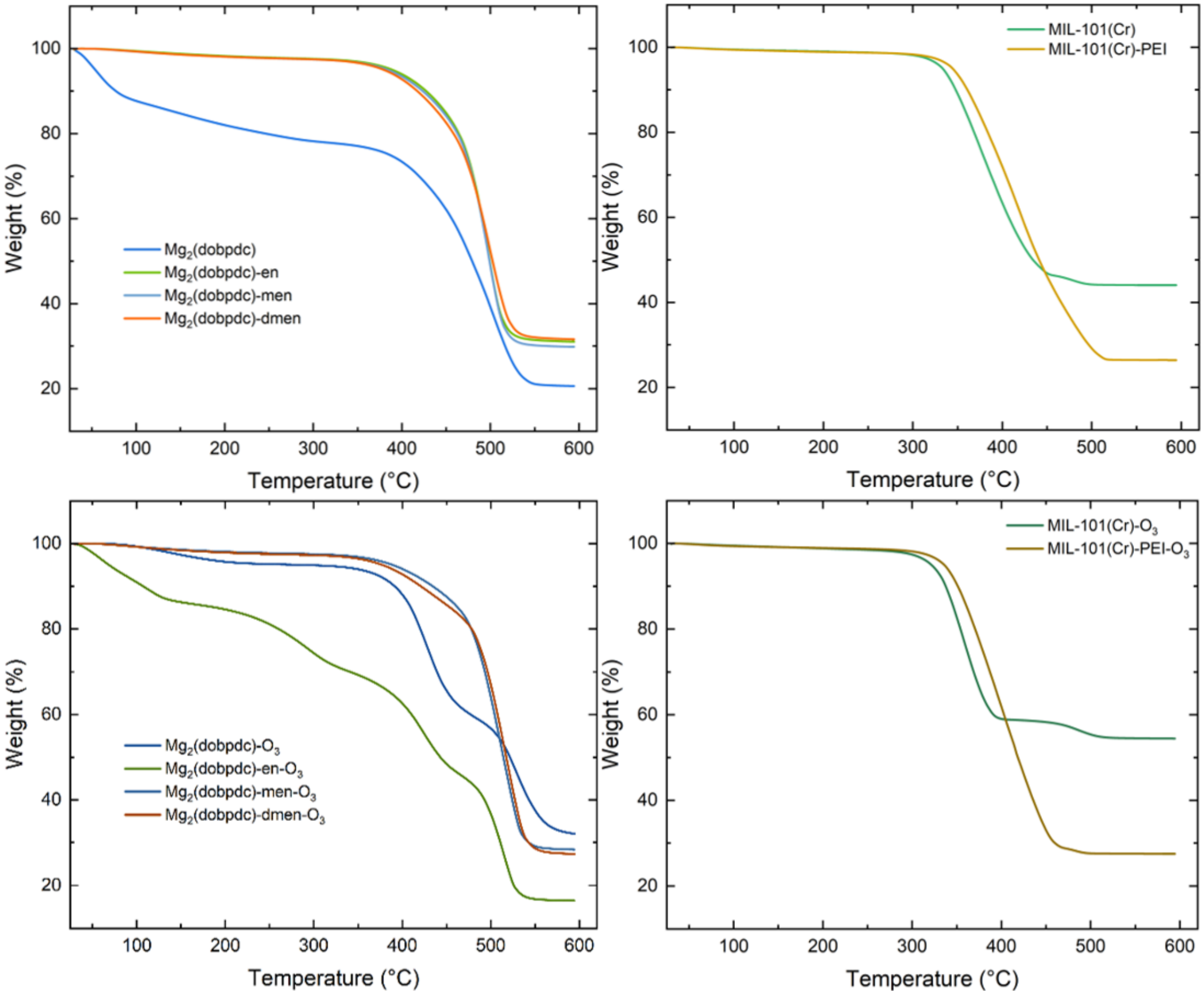

Figure S51. Thermogravimetric analyses of Mg_2(dobpdc) and MIL-101(Cr) bare MOF and amine composite samples before and after exposure to high concentrations of ozone. Mg_2(dobpdc) MOF and Mg2(dobpdc)-en show a change from one tranisiton with onset between 400-500 °C to two transitions, which is attributed to oxidation of ethylenediamine and potential oxidation of the MOF ligand. MIL-101(Cr) MOF and MIL-101(Cr)-PEI samples do not present any evident changes, which is attributed to the higher chemical stability of MIL-101(Cr).

References

(1) Darunte, L. A.; Oetomo, A. D.; Walton, K. S.; Sholl, D. S.; Jones, C. W. Direct Air Capture of CO2 Using Amine Functionalized MIL-101(Cr). *ACS Sustain. Chem. Eng.* **2016**, *4* (10), 5761–5768. https://doi.org/10.1021/acssuschemeng.6b01692.

(2) Carter, E. A.; Hungerford, J. T.; Joshi, J. N.; DeWitt, S. J. A.; Jiang, X.; Marszalek, B.; Lively, R. P.; Walton, K. S. Chemical Stability of MIL-101(Cr) upon Adsorption of SO_2 and NO_2 under Dry and Humid Conditions. *Ind. Eng. Chem. Res.* **2023**, *62* (22), 8864–8872. https://doi.org/10.1021/acs.iecr.3c00209.

(3) Niknam, E.; Panahi, F.; Daneshgar, F.; Bahrami, F.; Khalafi-Nezhad, A. Metal–Organic Framework MIL-101(Cr) as an Efficient Heterogeneous Catalyst for Clean Synthesis of Benzoazoles. *ACS Omega* **2018**, *3* (12), 17135–17144. https://doi.org/10.1021/acsomega.8b02309.
(4) Llewellyn, P. L.; Bourrelly, S.; Serre, C.; Vimont, A.; Daturi, M.; Hamon, L.; De Weireld, G.; Chang, J.-S.; Hong, D.-Y.; Kyu Hwang, Y.; Hwa Jhung, S.; Férey, G. High Uptakes of CO2 and CH4 in Mesoporous Metal—Organic Frameworks MIL-100 and MIL-101. *Langmuir* **2008**, *24* (14), 7245–7250. https://doi.org/10.1021/la800227x.
(5) Sheikh Alivand, M.; Hossein Tehrani, N. H. M.; Shafiei-alavijeh, M.; Rashidi, A.; Kooti, M.; Pourreza, A.; Fakhraie, S. Synthesis of a Modified HF-Free MIL-101(Cr) Nanoadsorbent with Enhanced H2S/CH4, CO2/CH4, and CO2/N2 Selectivity. *J. Environ. Chem. Eng.* **2019**, *7* (2), 102946. https://doi.org/10.1016/j.jece.2019.102946.
(6) Jamdade, S.; Cai, X.; Sholl, D. S. Assessment of Long-Term Degradation of Adsorbents for Direct Air Capture by Ozonolysis. *J. Phys. Chem. C* **2025**, *129* (1), 899–909. https://doi.org/10.1021/acs.jpcc.4c07054.
(7) Bhosale, G. S.; Vaidya, P. D.; Joshi, J. B.; Patil, R. N. Kinetics of Ozonation of Phenol and Substituted Phenols. *Ind. Eng. Chem. Res.* **2019**, *58* (18), 7461–7466. https://doi.org/10.1021/acs.iecr.9b00337.
(8) Bhattacharyya, S.; Han, R.; Joshi, J. N.; Zhu, G.; Lively, R. P.; Walton, K. S.; Sholl, D. S.; Nair, S. Stability of Zeolitic Imidazolate Frameworks in NO2. *J. Phys. Chem. C* **2019**, *123* (4), 2336–2346. https://doi.org/10.1021/acs.jpcc.8b11377.
(9) Biesinger, M. C. Accessing the Robustness of Adventitious Carbon for Charge Referencing (Correction) Purposes in XPS Analysis: Insights from a Multi-User Facility Data Review. *Appl. Surf. Sci.* **2022**, *597*, 153681. https://doi.org/10.1016/j.apsusc.2022.153681.
(10) Hueso, J. L.; Espinós, J. P.; Caballero, A.; Cotrino, J.; González-Elipe, A. R. XPS Investigation of the Reaction of Carbon with NO, O2, N2 and H2O Plasmas. *Carbon* **2007**, *45* (1), 89–96. https://doi.org/10.1016/j.carbon.2006.07.021.
(11) Hernandha, R. F. H.; Umesh, B.; Patra, J.; Tseng, C.-J.; Hsieh, C.-T.; Li, J.; Chang, J.-K. Double Nitrogenation Layer Formed Using Nitric Oxide for Enhancing Li+ Storage Performance, Cycling Stability, and Safety of Si Electrodes. *Adv. Sci.* **2024**, *11* (25), 2310062. https://doi.org/10.1002/advs.202310062.
(12) Justin, A.; Schertenleib, T.; Roth, J.; Espín, J.; Queen, W. L. Immobilizing Amine Species in a Cr-MOF for Enhanced Selectivity in CO2 Cycloaddition Reactions. *Chem. Commun.* **2025**, *61* (35), 6530–6533. https://doi.org/10.1039/D4CC05453A.
(13) Nam, H. Y.; Lee, G.; Jhung, S. H. Selective CO2 Adsorption over a Zr-Based Metal–Organic Framework Functionalized with Tris(2-Aminoethyl)Amine. *Chem. Eng. J.* **2024**, *494*, 153072. https://doi.org/10.1016/j.cej.2024.153072.
(14) Mohtasebi, A.; Chowdhury, T.; Hsu, L. H. H.; Biesinger, M. C.; Kruse, P. Interfacial Charge Transfer between Phenyl-Capped Aniline Tetramer Films and Iron Oxide Surfaces. *J. Phys. Chem. C* **2016**, *120* (51), 29248–29263. https://doi.org/10.1021/acs.jpcc.6b09950.
(15) La, Y.-H.; Kim, H. J.; Maeng, I. S.; Jung, Y. J.; Park, J. W.; Kim, K.-J.; Kang, T.-H.; Kim, B. Selective Cleavage of Nitro Groups in Nitro-Substituted Aromatic Monolayers by Synchrotron Soft X-Rays: Effect of Molecular Structure on Cleavage Rates. *Langmuir* **2002**, *18* (6), 2430–2433. https://doi.org/10.1021/la011208g.
(16) Ardizzone, S.; Bianchi, C. L.; Fadoni, M.; Vercelli, B. Magnesium Salts and Oxide: An XPS Overview. *Appl. Surf. Sci.* **1997**, *119* (3), 253–259. https://doi.org/10.1016/S0169-4332(97)00180-3.
